# Stabilized adaptive states in microbiome–human integrated physiology: reframing health and chronic disease as symbiotic biological states

**DOI:** 10.3389/fmed.2026.1824897

**Published:** 2026-05-14

**Authors:** João Francisco Pollo Gaspary, Luis Felipe Dias Lopes, Fernanda Peron Gaspary, Eduarda Grando Lopes, Alfred Lee Edgar, Eduardo Poletti Camara, Antonio Geraldo Camara

**Affiliations:** 1 Institute AuBento - Center for Education, Clinical Practice, and Research in Orthomolecular and Integrative Medicine, Santa Maria, Brazil; 2 Postgraduate Program in Administration and Accounting, Center for Social and Human Sciences, Federal University of Santa Maria, Santa Maria, Brazil; 3 Franciscan University, Santa Maria, Brazil; 4 Veterinary Medicine Course, Federal University of Santa Maria, Santa Maria, Brazil; 5 Department of Research and Development, ElastroCrete, LLC, Veyo, UT, United States; 6 Institute Camara - Center for Clinical and Orthomolecular Practice, Ribeirão Preto, Brazil; 7 Department of Cardiology and Internal Medicine, Beneficência Portuguesa de São Paulo, Ribeirão Preto, Brazil

**Keywords:** adaptive states, chronic disease stability, membrane-level signal integration, microbiome–host interaction, multigenomic physiology, systems physiology

## Abstract

**Background:**

Modern medicine has achieved remarkable precision in identifying molecular mechanisms and developing targeted interventions. However, a persistent clinical paradox remains: many chronic conditions—including metabolic, autoimmune, neuropsychiatric, and oncological disorders—exhibit long-term stability, resistance to guideline-concordant treatment, and recurrent trajectories. Despite extensive mechanistic characterization, the organizational basis of this stability remains insufficiently explained.

**Conceptual gap:**

In acute contexts such as infection and environmental intoxication, organisms can remain internally coherent while temporarily prioritizing non-host biological demands. This state-based perspective, however, has rarely been extended to chronic disease. At the same time, microbiome research has demonstrated that human physiology operates within a multigenomic system, in which exogenous gene repertoires contribute substantial metabolic and signaling capacity. Epigenetic research further indicates that repeated ecological exposures can progressively stabilize adaptive biological states over time.

**Proposed framework:**

We propose a conceptual framework in which health and disease are interpreted as stabilized adaptive states emerging from hierarchical signal integration within a multigenomic human system. In this model, chronic pathology reflects coherent but constrained regulatory configurations, rather than simple dysregulation or isolated system failure. Central to this interpretation is membrane-level decisional architecture, which governs signal routing, threshold modulation, and downstream transcriptional responses across tissues.

**Implications:**

This framework reorganizes existing evidence into a systems-level interpretation of chronic disease stability, providing a basis for generating testable hypotheses regarding state transitions, responsiveness to perturbation, and restoration of physiological flexibility. Rather than introducing new therapeutic doctrines, the model aims to clarify how biological systems stabilize over time and how such stabilization may be investigated within existing experimental paradigms.

**Systematic review registration:**

https://www.crd.york.ac.uk/PROSPERO/view/CRD420261295889, CRD420261295889; https://www.crd.york.ac.uk/PROSPERO/view/CRD420261295945, CRD420261295945.

## 1 Introduction

Over the past decades, modern medicine has achieved extraordinary precision in identifying molecular mechanisms, signaling cascades, and genetic determinants underlying human disease. Yet across medical specialties, clinicians frequently encounter chronic conditions that remain partially responsive or refractory to guideline-concordant interventions, often exhibiting relapse despite mechanistically targeted therapy. While molecular pathways are increasingly well characterized, the organizational basis of such long-term therapeutic refractoriness remains incompletely clarified (1–3). This recurrent pattern raises the possibility that chronic physiological stability may not be fully explained by isolated pathway dysfunction alone.

In parallel, microbiome research has demonstrated extensive and persistent participation of resident microbial communities in human physiology (4–7). Microbial metabolites, structural components, and genomic repertoires influence metabolic routing, immune calibration, neuroendocrine signaling, barrier integrity, and redox balance across multiple anatomical domains. These findings have significantly expanded understanding of host–microbe interdependence and ecological influence within human biology (7).

Despite this progress, microbial activity is predominantly framed as modulatory rather than structurally constitutive (5, 7). Human physiology and pathophysiology continue to be predominantly organized within host-genome–centered precision frameworks (8, 9), in which molecular stratification and biomarker-driven targeting define disease architecture and therapeutic logic (3). Within these models, microbial contributions are typically described as influence, interaction, or bidirectional signaling layered onto a primarily host-driven regulatory architecture. Although colonization of discrete anatomical niches is well documented, these domains are rarely synthesized into an overarching model of physiological organization.

Despite extensive mechanistic mapping across host and microbial domains (5, 7), integrative organizational synthesis remains comparatively limited. This asymmetry leaves unresolved a foundational question: how should persistent host–associated microbial coexistence be interpreted within long-term physiological stability? While ecological interdependence is increasingly acknowledged (7), the implications of sustained microbial–human interaction for the structural organization of chronic physiological states remain under examined.

Although systems biology has long emphasized network-level regulation in biological systems (10), the organizational determinants through which physiological states stabilize over time remain comparatively under-integrated in contemporary pathophysiological reasoning. This gap becomes particularly visible in chronic disease contexts, where long-term physiological stability and therapeutic refractoriness coexist with increasingly detailed molecular knowledge.

The present work therefore evaluates whether patterns of chronic stability and therapeutic refractoriness can be more coherently interpreted through structured integrative synthesis across host–exogenous biological interaction layers and membrane-level regulatory dynamics. Rather than introducing new molecular mechanisms, this work proposes that persistent chronic states can be more coherently interpreted as stabilized organizational regimes emerging from membrane-level signal prioritization within integrated multigenomic physiology.

## 2 Methods

To evaluate whether a multigenomic ecological perspective provides a more coherent explanatory architecture for long-term physiological states, a structured research design grounded in Work Breakdown Structure (WBS) logic (PMI, 2019, 2021) (11, 12) was implemented. This approach is consistent with a mechanistic integrative synthesis framework, in which structured evidence is reorganized to reveal cross-domain architectural coherence rather than aggregated for quantitative effect estimation.

**Figure 1:**
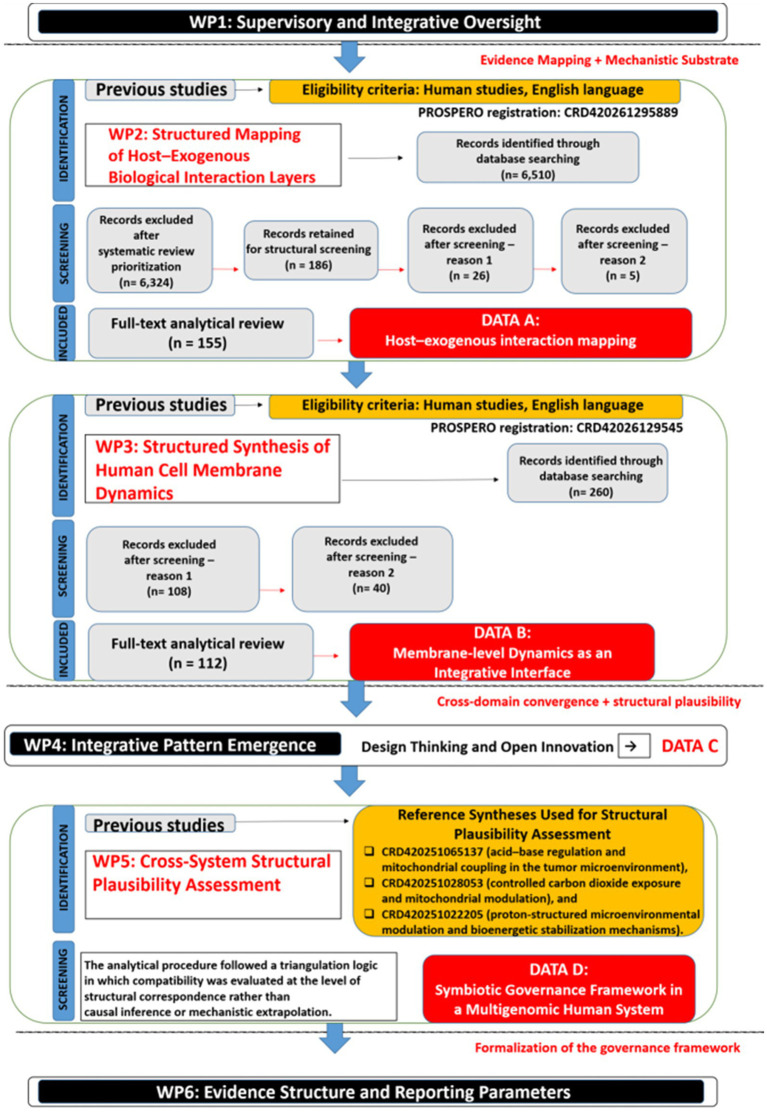
Structured analytical architecture of the study.

Note. The research design followed a Work Breakdown Structure (WBS) framework integrating systematic evidence mapping (WP2–WP3), cross-domain pattern convergence (WP4), structural plausibility calibration (WP5), and formal systems integration (WP6). The workflow progressively transforms distributed mechanistic evidence into a unified governance-based interpretation of stabilized physiological states in a multigenomic human system.

The WBS methodology enabled explicit partitioning of the research process into predefined Work Packages (WP1–WP7), ensuring strict separation between systematic evidence retrieval, mechanistic mapping, cross-domain validation, integrative consolidation, and framework formalization. This modular design minimized interpretative circularity, preserved inferential boundaries between analytical stages, and maintained transparency in the progression from empirical evidence to structural synthesis, as illustrated in Figure 1.

### 2.1 WP1—supervisory and integrative oversight

WP1 operated as a structural oversight layer, preserving conceptual coherence and methodological alignment across all work packages. This component ensured that evidence acquisition, analytical decomposition, and integrative interpretation remained sequential and non-overlapping.

Within this supervisory framework, a progressive search refinement logic was adopted where appropriate, enabling structured narrowing of evidentiary scope without compromising architectural representativeness. This process was inherently iterative at the level of scope refinement and cross-domain alignment, while preserving strict inferential separation between analytical stages.

In addition, structured cross-disciplinary consultation, consistent with open innovation principles (13), was incorporated to challenge interpretative bias and preserve inferential transparency. WP1 did not introduce independent datasets but functioned as a procedural stabilizer to maintain epistemic discipline across stages.

### 2.2 WP2—structured mapping of host–exogenous biological interaction layers

WP2 consisted of a prospectively registered systematic synthesis PROSPERO (14) (CRD420261295889) designed to map structured layers through which exogenous genomic material interacts with human physiological systems. Rather than treating host–microbe interactions as a homogeneous evidentiary field, the synthesis was organized *a priori* into three predefined analytical layers reflecting increasing levels of biological integration.

#### 2.2.1 Search strategy

Search architecture was structured according to three predefined analytical layers.

Layer I (Functional Integration) included descriptors targeting established physiological domains in which host–microbe interactions are extensively documented, including immune regulation, endocrine signaling, neurochemical modulation, barrier function, and metabolic–redox processes.

Layer II (Regulatory Interface) comprised descriptors addressing membrane-level signaling, redox modulation, psychoneuroendocrine integration, and adaptive response conditioning.

Layer III (Explicit Genetic-Level Interaction) included descriptors targeting direct exogenous DNA and RNA interactions within human physiological contexts.

This layered search structure enabled systematic differentiation between domain-level integration, regulatory-interface mediation, and direct genetic interaction prior to evidence synthesis. For the purposes of this structured mapping, the term “microbiome” was operationally defined to encompass bacterial, fungal (mycobiome), archaeal, and protozoan communities residing within human-associated ecological niches, unless otherwise specified by descriptor refinement.

The objectives of WP2 were to assess the extent of domain-level consolidation within functional host–microbe interactions and to evaluate the presence of explicit genetic interaction literature within the broader evidentiary landscape. The distribution of descriptors and the corresponding number of records identified and retained for structural synthesis are summarized in Table 1.

**Table 1:** Structured descriptor distribution and retention across predefined integration layers.

Layer	Descriptor combination	Records identified	Selected for structural synthesis
I – Functional Integration			
	microbiome AND “human physiology”	415	9
	microbiome AND “human host”	491	1
	microbiome AND “neuroendocrine”	394	7
	microbiome AND “immune regulation”	767	6
	microbiome AND “immune modulation”	519	1
	microbiome AND “metabolic regulation”	227	2
	microbiome AND “barrier function”	1981	12
	microbiome AND “mitochondrial function”	256	4
Layer I Total		5,050	42
II – Regulatory Interface			
	microbiome AND “psychoneuroendocrine”	3	1
	microbiome AND “endocrine signaling”	17	10
	microbiome AND “redox regulation”	9	3
	microbiome AND “membrane signaling”	2	1
	parasite AND “human physiology”	142	0
	parasite AND “human host”	1,103	7
	virome AND “human host”	19	14
	virome AND “human physiology”	12	8
	(parasite OR microbiome OR virome) AND “adaptive response”	78	65
Layer II Total		1,385	109
III – Explicit Genetic-Level Interaction			
	(“exogenous DNA” OR “exogenous RNA” OR “viral RNA”) AND “human host”	74	34
	“microbial small RNAs” AND “human host”	1	1
Layer III Total		75	35
Overall Total		6,510	186

#### 2.2.2 Selection logic

Duplicate records across descriptor combinations were removed prior to structural consolidation. Retention counts reflect unique studies meeting predefined eligibility criteria.

A substantial reduction from 6,510 identified records to 186 retained studies resulted primarily from prioritizing systematic reviews and meta-analyses as integrative evidence units wherever available, thereby avoiding redundancy across highly synthesized domains.

Under this logic, when a systematic review or meta-analysis was identified within a given descriptor cluster, primary studies addressing the same mechanistic construct were not retained individually, as their evidentiary content was considered structurally represented within the integrative synthesis. This approach was adopted to avoid redundancy and to maintain a structurally balanced evidence architecture across integration layers.

This contraction also reflects the distinction between descriptive publication density and structurally relevant evidence. The search architecture was intentionally broad to map the full evidentiary landscape across integration layers; however, many records within high-density domains (e.g., barrier function, immune regulation) represented mechanistically overlapping syntheses or descriptive expansions of already consolidated constructs. WP2 did not aim for thematic exhaustiveness within each descriptor cluster, but for structural representativeness across predefined integration layers. Retention therefore prioritized studies that introduced distinct mechanistic constructs relevant to adaptive stabilization, rather than studies that reiterated established domain-level associations. This architectural approach explains the marked quantitative contraction between records identified and studies retained for structural synthesis.

When systematic reviews or meta-analyses were available within a descriptor cluster, these were preferentially retained as integrative evidence units, given their consolidated representation of mechanistic domains. In contrast, for descriptor combinations in which no formal synthesis existed—particularly within the Regulatory Interface and Explicit Genetic Interaction layers—high-quality mechanistic primary studies were retained to prevent structural under-representation of emergent or under-synthesized domains. Thus, retention criteria varied according to evidentiary maturity within each layer, balancing consolidation where literature was dense and mechanistic diversity where synthesis was sparse. This approach ensured proportional structural mapping rather than uniform numerical sampling across search rotors.

Following Round 1 selection (*n =* 186), a second-stage structural refinement was conducted to optimize architectural representativeness and minimize redundancy across descriptor domains. This process reduced the dataset to 155 unique studies.

Round 2 refinement consisted of removing overlapping systematic reviews covering identical mechanistic domains and excluding studies that did not contribute additional structural variation beyond constructs already retained. Throughout this process, proportional coverage across the three predefined analytical layers was preserved to prevent under-representation of domains characterized by lower publication density but high mechanistic specificity.

This reduction was undertaken as an architectural optimization step rather than as evidentiary filtration, ensuring that subsequent cross-WP integration reflected structural diversity and inferential balance rather than repetition density.

**Figure 2:**
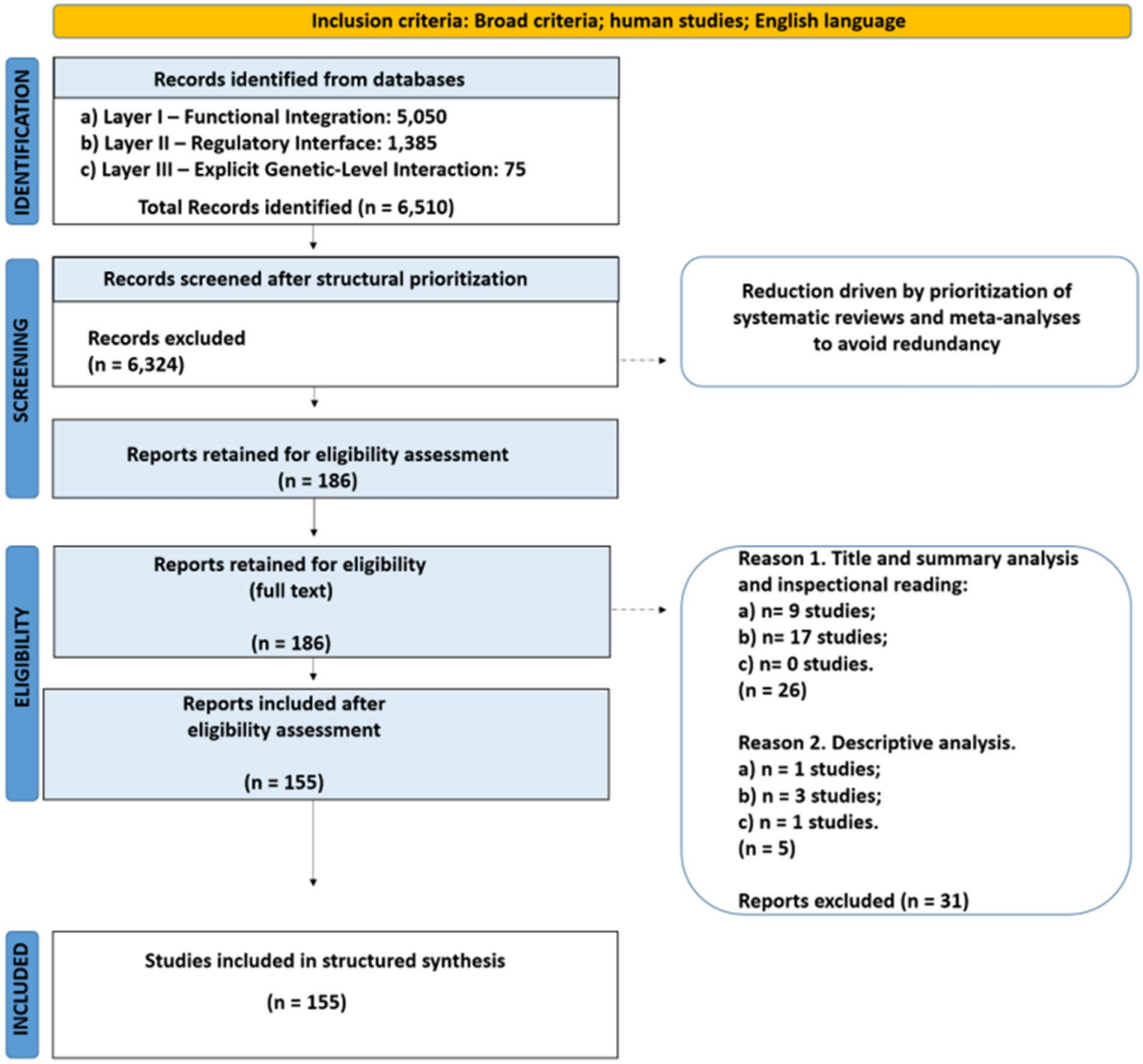
PRISMA-based flow diagram for WP2 study selection.

The resulting 155-study corpus constituted the structured evidentiary substrate for subsequent cross-domain integration and membrane-level evaluation in WP3 and WP4. Study selection and structural refinement were conducted within a prospectively registered framework PROSPERO (14) (CRD420261295889), ensuring that inclusion criteria and analytical structure were defined *a priori* and consistently applied throughout the refinement process. The study selection process was conducted under prospectively defined criteria and is summarized in a PRISMA-based flow diagram (Figure 2).

### 2.3 WP3—structured synthesis of human cell membrane dynamics

WP3 consisted of a prospectively registered systematic review PROSPERO (14) (CRD42026129545) designed to examine whether membrane-level organizational variables constitute a regulatory substrate through which exogenous signals acquire functional relevance within human cellular systems.

Whereas WP2 mapped host–exogenous interaction layers across physiological domains, WP3 focused specifically on membrane-level dynamics as an integrative interface. The objective was to isolate mechanistic evidence linking membrane organization to signal routing, threshold modulation, and state-dependent cellular responsiveness.

The review targeted studies addressing membrane-dependent signal routing, lipid microdomain organization and receptor clustering, electrochemical gradient dynamics, redox–lipid coupling, state-dependent responsiveness, and non-linear cellular behavior.

#### 2.3.1 Search strategy

Searches were conducted in PubMed/MEDLINE from database inception to the most recent available date using predefined descriptor clusters aligned with membrane-level regulatory constructs. Filters were restricted to English-language publications and human-relevant experimental models where applicable.

A structured rotor architecture was implemented to progressively narrow from global membrane dynamics to specific nucleic-acid routing competence. Three analytical rotors were defined.

Rotor I targeted electrochemical membrane properties associated with adaptive responsiveness and state-dependent behavior, yielding 185 records prior to deduplication.

Rotor II focused on cholesterol-enriched microdomain organization and pattern recognition receptor recruitment, yielding 58 records prior to deduplication.

Rotor III refined the search to studies demonstrating membrane-dependent uptake, adaptor translocation, and routing of nucleic acids to signal-permissive compartments, yielding 17 records prior to deduplication.

Full descriptor combinations and record distribution are presented in Table 2.

**Table 2:** Structured rotor architecture and study retention across membrane-level analytical axes.

Analytical axis	Descriptor combination	Records identified	Studies retained
Electrochemical State–Responsiveness	(“membrane potential”) AND (adaptation OR homeostasis OR regulation) AND (hysteresis OR “nonlinear response” OR bistability OR plasticity)	185	81
Microdomain–PRR Recruitment	(“TLR4” OR “TLR3” OR “TLR7” OR “TLR8” OR “TLR9”) AND (“lipid raft” OR caveolae OR “membrane microdomain”) AND (“RNA” OR “DNA” OR “nucleic acid”)	58	24
Nucleocapture–Routing Interface	Refined subset emphasizing uptake, adaptor translocation, and compartmental delivery	17	7
Total		260	112

#### 2.3.2 Selection logic

Initial retrieval across all three rotors yielded 260 records prior to deduplication. Selection proceeded in two structured stages.

Importantly, retention across rotors was not proportional to raw retrieval counts but to structural density and mechanistic diversity within each analytical axis. Rotor I captured broader electrochemical and state-dependent membrane phenomena and therefore required wider representational coverage to preserve variability in threshold modulation and persistence models. Rotor II functioned as a bridging axis, focusing on cholesterol-dependent microdomain organization and receptor recruitment; retention within this rotor was calibrated to capture distinct mechanistic paradigms of lipid-mediated signal amplification without over-representing repetitive TLR-centric models. Rotor III targeted highly specific nucleocapture–routing mechanisms, where mechanistic redundancy was frequent and a smaller number of studies sufficiently captured routing-dependent structural variation. Retention was therefore guided by saturation of mechanistic diversity rather than numerical symmetry across rotors, ensuring balanced architectural representation across electrochemical, microdomain, and routing-dependent determinants of signal competence.

Stage 1 consisted of mechanistic relevance filtering. Studies were retained if they demonstrated experimentally verified membrane-dependent signal routing, inducible receptor recruitment into lipid microdomains, cholesterol-dependent modulation of signaling amplitude, or routing-dependent transcriptional competence. Descriptive lipid raft reviews lacking functional linkage to signaling competence and studies focused solely on therapeutic outcomes without membrane organizational variables were excluded.

Stage 2 involved structural redundancy minimization. Conceptual overlap across rotors was evaluated and duplicative mechanistic demonstrations were consolidated. Reduction from 260 to 112 retained studies reflected removal of overlapping receptor models, exclusion of peripheral descriptive studies, and preservation of proportional representation across all predefined axes.

**Figure 3:**
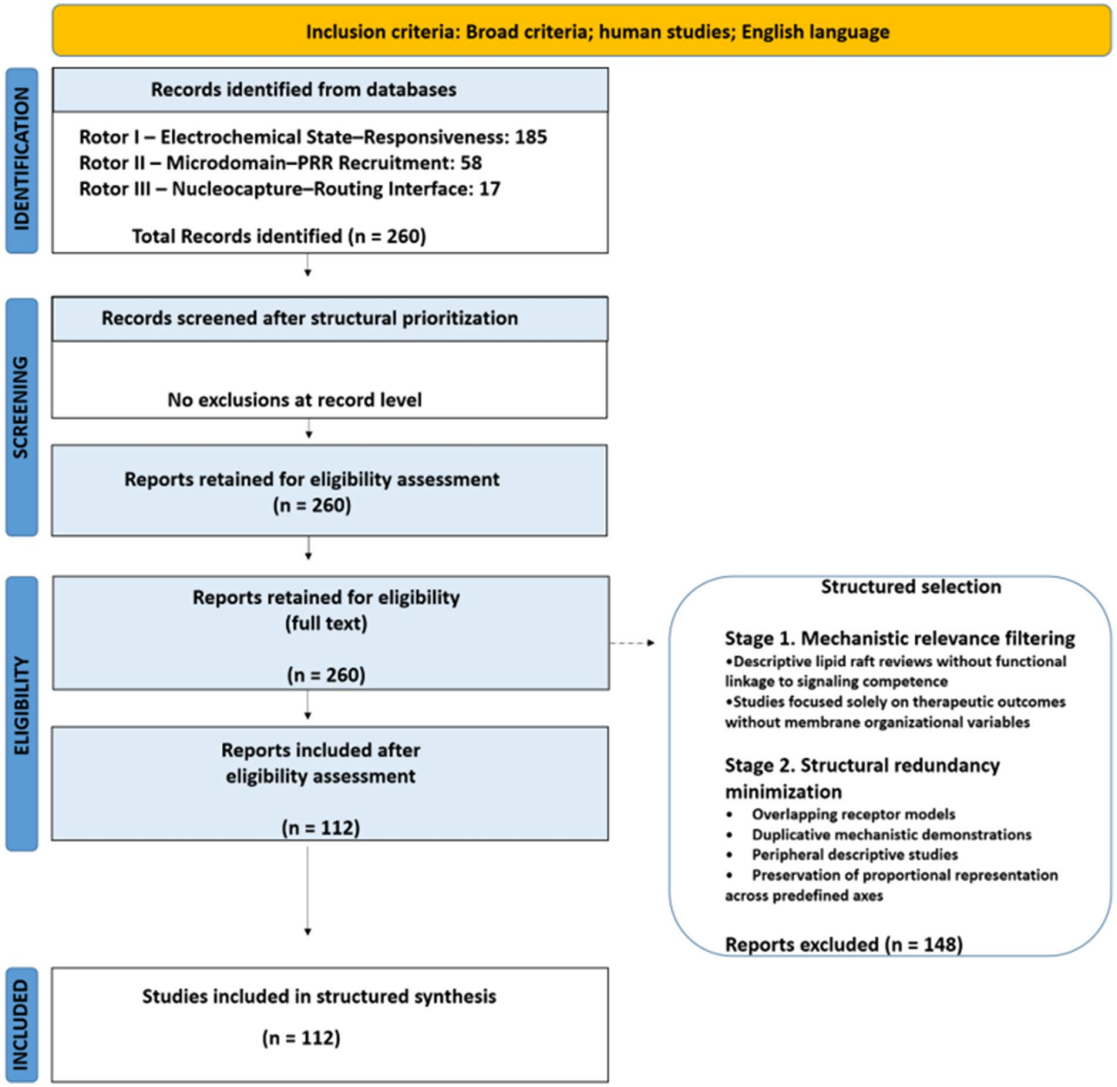
PRISMA-based flow diagram for WP3 study selection.

The final dataset of 112 retained studies constituted the structured mechanistic corpus for subsequent integrative analysis. Importantly, this selection and refinement process was conducted within a prospectively registered framework PROSPERO (14) (CRD420261295945), with inclusion guided by predefined criteria related to physiological relevance, membrane-level focus, and contribution to state-dependent cellular understanding, while refinement steps were applied consistently throughout the synthesis process. The study selection process followed a structured, mechanism-oriented workflow and is summarized in a PRISMA-based flow diagram (Figure 3).

### 2.4 WP4—integrative pattern emergence

WP4 constituted a structured integrative synthesis designed to consolidate the mechanistic findings generated in WP2 and WP3 through a formal cross-domain analytical framework. Rather than introducing new empirical datasets, this stage functioned as a convergence layer applying predefined interpretative criteria to previously extracted constructs.

The integrative process was deliberately iterative. Mechanistic variables derived from WP2 and WP3 were first decomposed into operational descriptors without hierarchical assumptions, enabling analytical expansion across domains prior to consolidation. These descriptors included immune setpoint modulation, barrier gating dynamics, redox–metabolic conditioning, microdomain recruitment patterns, nucleic-acid routing competence, and lipid remodeling variables.

The integrative phase followed a structured divergence–convergence logic consistent with Design Thinking principles (15), enabling exploratory recombination of mechanistic variables prior to disciplined consolidation under predefined criteria. This process was conducted within an Open Innovation framework, incorporating iterative cross-disciplinary dialogue while preserving methodological traceability and explicit inferential boundaries.

Subsequently, structured cross-domain alignment was performed to evaluate structural correspondences between membrane-level determinants of responsiveness and host–exogenous interaction layers. Convergence was permitted only when independently derived constructs demonstrated operational coherence within human cellular physiology and remained confined to the empirical scope of WP2 and WP3.

Emergent structural correspondences were retained under predefined stringency thresholds. Structures were required to be supported by mechanistic evidence across at least two analytical domains and to demonstrate compatibility with experimentally grounded membrane-level variables. Interpretative expansion beyond the evidentiary limits of prior work packages was explicitly avoided.

WP4 therefore established the procedural conditions under which higher-order interpretative constructs could emerge in the Results section. The integrative architecture subsequently articulated reflects disciplined cross-domain convergence rather than externally imposed theoretical expansion.

### 2.5 WP5—cross-system structural plausibility assessment

WP5 was designed as a cross-system structural plausibility assessment. Its purpose was to examine whether the structural correspondences identified through cross-domain integration in WP4 remained internally coherent when evaluated against well-characterized microenvironment-dependent stabilization phenomena described in independent physiological contexts.

This stage did not seek to establish generalizability, quantify effect sizes, or introduce new empirical evidence. Instead, it functioned as a bounded consistency check, evaluating whether the integrative constructs derived from WP2–WP4 were structurally compatible with recognized mechanisms of durable physiological reconfiguration arising from physicochemical and microenvironmental constraints.

The assessment emphasized stabilization-relevant variables including acid–base modulation, proton dynamics, redox tone, membrane-level integration, and mitochondrial coupling efficiency, as these represent plausible cross-domain substrates influencing threshold behavior and state persistence.

Methodologically, WP5 drew upon previously registered systematic reviews addressing microenvironment-dependent regulation and bioenergetic stabilization, including PROSPERO (14) CRD420251065137 (acid–base regulation and mitochondrial coupling in the tumor microenvironment), CRD420251028053 (controlled carbon dioxide exposure and mitochondrial modulation), and CRD420251022205 (proton-structured microenvironmental modulation and bioenergetic stabilization mechanisms). These registrations were used strictly as structured reference frameworks to guide cross-domain comparison, rather than as extensions of the evidentiary base established in WP2–WP3. Their function was to provide calibration anchors during structural alignment, thereby maintaining inferential discipline and minimizing interpretative drift.

The analytical procedure followed a triangulation logic in which compatibility was evaluated at the level of structural correspondence rather than causal extrapolation. No additional mechanisms were introduced, and no claims beyond the empirical boundaries of prior work packages were inferred.

WP5 therefore functioned as a boundary calibration stage, establishing structural coherence under physicochemical constraint prior to formal systems consolidation in WP6.

### 2.6 Evidence structure and reporting parameters

The systematic components of this study followed structured PRISMA-informed parameters (16) adapted to the multi-layered WBS architecture. The systematic syntheses informing WP2 and WP3 were prospectively registered in PROSPERO (14) to ensure methodological transparency and traceability.

Eligibility criteria prioritized mechanistic or functional evidence demonstrating physiologically relevant host–exogenous interactions across immune, endocrine, neural, metabolic, and barrier-related domains. Purely taxonomic descriptions, technical microbiome reports lacking physiological integration, and studies restricted to acute lethality without adaptive context were excluded. Animal and *in vitro* studies were retained only when providing mechanistic insight with translational relevance to human physiology.

Literature searches were conducted in PubMed/MEDLINE, Embase and Web of Science using structured descriptor clusters aligned with predefined analytical layers. Selection followed a two-stage filtering process emphasizing mechanistic coherence and structural representativeness rather than publication type alone.

Data extraction focused on biological interface, exogenous genetic source class, host systems involved, and adaptive stabilization features. Rather than aggregating effect sizes, synthesis prioritized identification of recurrent structural patterns across domains.

Given the integrative and cross-domain nature of the study, formal quantitative risk-of-bias instruments designed for intervention trials were not applied. Instead, methodological rigor was assessed qualitatively through transparency, cross-domain consistency, biological plausibility, and acknowledgment of known limitations such as low-biomass contamination in microbiome research.

Extraction and consolidation of higher-order constructs (FPV’s, CSF’s, and Mechanistic Indicator Sets) during WP2 and WP3 synthesis followed a structured qualitative multi-criteria decision logic (17, 18). Criteria included cross-domain convergence, mechanistic distinctiveness, recurrence across independent evidence clusters, and compatibility with predefined integration layers. No quantitative weighting matrix was implemented; rather, construct retention was governed by architectural coherence and inferential stability across domains.

### 2.7 WP6—formal systems integration and translational structuring

WP6 integrated the structured evidence base generated in WP2 and WP3, the cross-system structural plausibility calibration conducted in WP5, and the disciplined cross-domain convergence established in WP4 into a formalized systems-level architecture. No new empirical datasets were introduced at this stage. Rather, WP6 synthesized previously validated constructs into a coherent multiscale framework confined to the inferential boundaries established throughout the preceding work packages.

The structural domains formalized in this stage were not introduced *a priori* but were derived through progressive abstraction of recurrent cross-domain stabilization variables identified during cross-domain integration. These domains reflect clustered patterns of convergence observed across membrane-level dynamics, bioenergetic modulation, and systemic stabilization features, as identified through integrative alignment of WP2 and WP3 within WP4.

To ensure translational coherence and research tractability, the resulting architecture was evaluated using established SMART (19) and FINER (20) criteria. The framework was assessed for specificity, measurability, feasibility, relevance, novelty, ethical neutrality, and operational clarity within contemporary biomedical research constraints. This evaluation did not constitute empirical validation but functioned as a structured appraisal of conceptual viability and methodological rigor.

#### 2.7.1 Derivation of operational hypotheses

The integrated architecture derived from WP2–WP5 was subsequently translated into operational hypotheses to enhance experimental tractability while preserving inferential discipline. This process is consistent with a mechanistic integrative synthesis approach, in which structured evidence is reorganized to reveal cross-domain architectural coherence rather than aggregated for quantitative effect estimation. This step did not introduce new empirical claims but consisted of a structured derivation process grounded in recurrent mechanistic correspondences identified across integration layers.

Operational hypothesis derivation followed a deductive logic constrained by three criteria: (i) cross-domain convergence of mechanistic variables, (ii) compatibility with experimentally validated membrane-level dynamics, and (iii) consistency with bioenergetic and physicochemical constraints established in WP5. Only constructs satisfying these conditions were translated into testable propositions, ensuring that hypothesis generation remained fully bounded within the evidentiary substrate established in prior work packages.

Rather than focusing on isolated molecular pathways, operationalization targeted system-level variables reflecting state-dependent physiological organization. These included, but were not limited to: (i) membrane-level signal routing competence, (ii) cholesterol-dependent microdomain recruitment and clustering dynamics, (iii) threshold-dependent modulation of receptor responsiveness, (iv) oscillatory range and reversibility of metabolic and inflammatory states, and (v) persistence of adaptive configurations following removal of initiating stimuli.

Within this framework, hypotheses are formulated as relationships between identifiable structural determinants and measurable physiological outcomes. For example, modulation of membrane lipid composition is expected to alter signal prioritization and downstream transcriptional amplitude independent of ligand availability; similarly, bioenergetic constraints affecting NAD^+^ availability and redox balance are expected to influence the reversibility and stability of physiological states.

Importantly, these hypotheses are not intended as predictive models in isolation but as experimentally addressable expressions of the integrated framework. Their function is to bridge conceptual architecture and empirical investigation by enabling the design of studies focused on state transitions, responsiveness to perturbation, and restoration of adaptive range.

This operational layer therefore represents a constrained translation of the integrative synthesis into testable research directions, maintaining explicit separation between evidence consolidation (WP2–WP3), cross-domain convergence (WP4), structural plausibility assessment (WP5), and hypothesis generation as a final, bounded step within formal systems integration.

WP6 therefore represents the formal consolidation layer of the WBS design (11, 12). It transforms cross-domain structural correspondence into an operationally bounded systems framework while preserving explicit inferential discipline.

## 3 Results

In accordance with the supervisory and integrative oversight defined in WP1, results are organized sequentially to mirror the analytical architecture under which evidence was retrieved and consolidated. This alignment ensures that data presentation remains structurally consistent with the predefined inferential boundaries of the research design.

This section presents consolidated outputs from WP2–WP4 as structured evidence patterns. Interpretative plausibility calibration (WP5) and formal systems consolidation (WP6) are addressed in subsequent sections to preserve analytical separation between evidence structuring and higher-order architectural synthesis.

### 3.1 Results of WP2—structural mapping of host–exogenous integration

WP2 generated a structured evidentiary corpus intended to characterize how host–exogenous interactions are represented across distinct physiological domains and levels of biological integration. The results presented in this section describe the consolidation of retrieved literature, the reorganization of evidence into higher-order interpretative axes, and the identification of recurrent mechanistic patterns emerging from cross-domain synthesis. No theoretical constructs are introduced at this stage; findings are confined to the structural properties of the consolidated corpus.

#### 3.1.1 Descriptor architecture and evidence consolidation

The WP2 search strategy identified 318 records across 19 descriptor combinations (pre-deduplication), structured according to three predefined analytical layers: Functional Integration, Regulatory Interface, and Explicit Genetic-Level Interaction.

Following Round 1 screening, which applied predefined eligibility criteria emphasizing physiological integration, translational relevance, and adaptive stabilization, 186 articles were retained. Systematic reviews and meta-analyses were prioritized where available. In domains lacking formal synthesis, high-quality mechanistic primary studies were retained to preserve structural completeness.

A second-stage refinement (Round 2) focused on cross-descriptor redundancy and marginal mechanistic contribution. This process reduced the consolidated corpus to 155 unique studies for cross-WP integration while preserving representation across all predefined analytical layers.

Although the search architecture was organized according to predefined layers, consolidation of the retained corpus revealed a reorganization of evidence into higher-order interpretative axes that did not mechanically replicate the initial stratification.

Table 3 presents the consolidation of evidence, which reorganized the corpus along stabilization-relevant interpretative axes rather than reproducing the original descriptor stratification. The FPVs represent emergent structural orientations derived from cross-domain integration rather than predefined analytical categories.

**Table 3:** Structural reframing of WP2 consolidated corpus using fundamental points of view (FPV’s).

FPV	Structural axis	Conceptual definition	Authors
FPV I	Functional Coupling Stabilization	Persistent bidirectional physiological coupling between host systems and resident microbial communities under adaptive constraint	Christmann et al. (75), Iliev and Cadwell (21), Zhou et al. (22), and Zhu et al. (76)
FPV II	Interface-Level Adaptive Conditioning	Membrane-mediated and microenvironment-dependent modulation of signaling thresholds under ecological stressors	Schroeder et al. (24), Abrescia et al. (25), and Armand et al. (77)
FPV III	Genetic-Level Signal Incorporation	Explicit molecular interaction between host and exogenous genetic material influencing regulatory competence	Anderson and Seifert (27), Simões-Barbosa et al. (78), and He et al. (28)

#### 3.1.2 Consolidation gradient

The distribution of evidence across layers revealed marked asymmetry in the maturation of the field. Functional microbiome–host integration is extensively consolidated through systematic syntheses across immune, barrier, metabolic, and neuroendocrine domains (21–23). In contrast, regulatory interface domains—including membrane-level integration and redox signaling (24–26) —remain minimally synthesized. Most notably, explicit exogenous DNA/RNA interaction with human physiology lacks formal systematic consolidation, despite emerging molecular evidence (27, 28).

This structural gradient suggests differential consolidation across analytical axes, indicating that functional host–microbe integration is extensively synthesized, whereas regulatory-interface and explicit genetic-interaction domains remain comparatively less consolidated in the current literature.

#### 3.1.3 Hierarchical distribution of evidence across integration layers

Evidence distribution revealed a pronounced structural gradient across the three FPV’s.

FPV I (Functional Coupling Stabilization) demonstrated robust consolidation. Domains such as immune modulation, barrier function, metabolic regulation, mitochondrial dynamics, and neuroendocrine interaction were frequently supported by systematic syntheses, indicating maturity at organ-system and physiological-network levels.

FPV II (Interface-Level Adaptive Conditioning) exhibited lower synthesis density. Although mechanistic evidence addressing membrane signaling, redox modulation, and adaptive response stabilization was present, formal integrative consolidation was limited.

FPV III (Genetic-Level Signal Incorporation) showed the lowest degree of systematic synthesis. Evidence for exogenous DNA/RNA participation existed but remained fragmented and rarely consolidated through integrative frameworks.

This hierarchical distribution reflects differential depth of mechanistic integration across analytical layers within the consolidated corpus, revealing structural asymmetry between domain-level synthesis and interface- or gene-level organizational mapping.

#### 3.1.4 Recurrent mechanistic convergence

To prevent descriptor-driven circularity, mechanistic stabilization constructs were not defined *a priori*. Instead, they were inductively derived from cross-domain convergence across the 155 retained records.

Table 4 formalizes the emergent Critical Success Factors (CSF’s) identified through this inductive process.

**Table 4:** Emergent critical success factors (CSFs) derived from mechanistic convergence.

Mechanistic construct (CSF)	Functional meaning	Representative evidence clusters	Authors
Barrier Plasticity	Dynamic modulation of epithelial permeability and structural integrity	IBD, SBS, astronaut gut, colitis	Iliev and Cadwell (21), Touchefeu et al. (79), and Akinsuyi et al. (80)
Immune Recalibration	Context-dependent tuning of innate–adaptive balance	Schistosoma, Candida, chronic pain	Afful et al. (81), Yano et al. (82), and Goudman et al. (83)
Metabolic Reprogramming	Host or microbial shifts in redox, lipid, or energy pathways	Indole, taurine, mitochondria	Armand et al. (77), Sánchez-Quintero et al. (26), and Pires et al. (84).
Neuroendocrine Coupling	Integration of microbial signals into HPA, vagal, or hormonal axes	Gut–brain axis, PSD, MS	Ahmed et al. (85), Dicks et al. (86), Barrio et al. (87), and Dos Santos and Galiè (88)
Ecological Selection Pressure	Host–microbe co-adaptation under environmental constraints	Hypoxia, altitude, PAHs	Zhu et al. (76), Sturgess and Montgomery (89), and Roslund et al. (90).
Genomic Exchange and Editing	Host sensing or modification of exogenous nucleic acids	APOBEC editing, small RNAs	Anderson and Seifer (27), He et al. (28), and Timmerman et al. (91).
Virome-Mediated Network Modulation	Phage and viral control of microbial–immune equilibrium	Anelloviruses, Caudoviricetes	Liang and Bushman (23), Widder et al. (92), Gulyaeva et al. (93), and Vietzen et al. (94)
Microbiota-Driven Signal Amplification	Microbial metabolites as systemic signaling amplifiers	SCFAs, bile acids, tryptophan metabolites	Armand et al. (77), Ahmed et al. (85), and Myhrstad et al. (95).

These CSF’s describe stabilization behaviors rather than isolated molecules. They represent system-level dynamics recurring across heterogeneous physiological contexts.

The asymmetrical density of evidence across FPV’s and CSF’s suggests that adaptive and ecological mechanisms are extensively characterized, whereas explicit genomic interface mechanisms remain comparatively under-integrated in current literature.

#### 3.1.5 Mechanistic indicator set

To enable structured cross-domain comparison without collapsing integration into single biomarkers, WP2 extracted a compact Mechanistic Indicator Set (MIS). This set reflects recurring operational readouts used in the literature to capture adaptive stabilization phenomena. Table 5 presents the MIS-WP2.

**Table 5:** Mechanistic indicator set extracted from WP2 (MIS-WP2).

Indicator	Descriptions	Typical readouts	Bridge to WP3/WP4	Representative authors
Immune setpoint reweighting	Sustained recalibration of innate–adaptive immune balance under chronic ecological exposure	Cytokine profiles; T helper 1/2/17 (Th1/Th2/Th17) and regulatory T cell (Treg) balance; pattern recognition receptor (PRR)-linked transcriptional signatures	Establishes threshold-dependent immune activation models informing membrane-level decisional dynamics (WP3)	Afful et al. (81), Yano et al. (82), and Vietzen et al. (94)
Barrier gating drift	Persistent modulation of epithelial selectivity and interface permeability under inflammatory or environmental constraint	Transepithelial electrical resistance (TEER); tight junction protein expression (e.g., claudins, occludin); permeability assays	Supports membrane-interface architecture as a selective decisional layer (WP3)	Iliev and Cadwell (21), Akinsuyi et al. (80), and Almutairi et al. (96)
Redox–gas modulation	Microenvironmental redox and gaseous constraints shaping signaling competence and adaptive thresholds	Reactive oxygen species markers; heme-dependent pathways; hypoxia-inducible signaling; oxidative stress indices	Converts metabolic flux into field-level regulatory constraints (WP3/WP4 integration)	Schroeder et al. (24), Armand et al. (77), and Sturgess and Montgomery (89)
Microbial metabolic field conditioning	Persistent modulation of host physiological environment through microbial metabolic outputs shaping systemic biochemical gradients.	Concentrations of short-chain fatty acids (SCFAs), secondary bile acids, tryptophan-derived metabolites, microbial fermentation products	Establishes ecological biochemical gradients that influence membrane-level signaling thresholds and metabolic routing competence (WP3/WP4 integration)	Valdes et al. (66), Zmora et al. (67), Fan and Pedersen (68), and Zhang et al. (69)
Metabolite-mediated crosstalk	Host receptor-mediated signaling responses triggered by microbiota-derived metabolites	Short-chain fatty acids (SCFAs); bile acids; indole derivatives; microbial metabolite profiling	Enables cross-domain pattern convergence during integrative synthesis (WP4)	Ahmed et al. (85), Myhrstad et al. (95), and Waterhouse et al. (66)
Exogenous nucleic-acid interface	Direct participation of microbial or viral nucleic acids in host regulatory processes	Extracellular vesicle-associated RNA; viral RNA sensing; transfer RNA-derived small RNAs; LINE-1 mediated events	Anchors explicit genomic-layer integration assessed in WP3	Anderson and Seifert (27), He et al. (28); and Timmerman et al. (91)
Genome mobility signals	Adaptive restructuring mediated by horizontal gene transfer and mobile genetic elements	Horizontal gene transfer (HGT); transposons; retrotransposons; hybridization signatures	Provides mechanistic plausibility for distributed genomic participation (WP4 extrapolation control)	Zhu et al. 2025 (76), Landeryou et al. 2022 (97), and Le Bihan et al. (98)
Species-jump constraints	Molecular determinants regulating host-range transitions and ecological host adaptation	Host restriction factors; viral host-range signatures; adaptation-associated genomic motifs	Ensures cross-interface coherence across ecological boundaries	Zhou et al. (22), Liang and Bushman (23), Mitri et al. (99), Handley (100), and Rampelli et al. (101).

The MIS does not constitute a diagnostic panel. Rather, it represents a convergent analytical vocabulary capturing recurrent stabilization features across ecological, molecular, and genomic interaction layers. These indicators provide a structured bridge between the functional integration patterns identified in WP2 and the membrane-level routing dynamics examined in WP3, thereby enabling disciplined cross-domain pattern emergence analysis in WP4.

Importantly, the Mechanistic Indicator Set (MIS) defines an intermediate observability layer within the WBS architecture, capturing recurrent system-level features through which multigenomic interactions become empirically traceable. Each indicator corresponds to measurable biological manifestations that preserve structural alignment with membrane-level dynamics (WP3) and support disciplined cross-domain pattern emergence in WP4.

In this context, the MIS does not introduce interpretative constructs but provides a structured vocabulary of observables that enables consistent mapping across analytical layers while preserving inferential separation between evidence extraction and subsequent integrative and translational stages.

#### 3.1.6 Structural interpretation

WP2 reveals four structural patterns within the consolidated corpus. First, microbiome–host integration is strongly consolidated at functional system levels, as evidenced by the predominance of systematic syntheses and high-density evidence clusters across immune, barrier, metabolic, and neuroendocrine domains identified in WP2. Second, regulatory-interface and explicit genetic-interaction layers remain under-synthesized. Third, recurrent cross-domain stabilization mechanisms are detectable through shared indicator families. Fourth, functional maturity of the field has outpaced ontological consolidation at membrane and genetic-interface levels.

This structural gradient established the evidentiary basis for WP3, which independently evaluated whether membrane-level dynamics provide a coherent integrative substrate through which exogenous genomic signals acquire decisional weight. The differential consolidation observed across FPVs indicates that while functional host–microbe coupling is extensively synthesized at organ-system levels, the regulatory-interface mechanisms through which such integration acquires operational decisional weight remain comparatively under-articulated. This structural gap provided the rationale for the focused membrane-level evaluation conducted in WP3.

### 3.2 Membrane-level determinants of signal competence

WP3 examined whether membrane-level organizational variables provide a mechanistic substrate through which host–exogenous interactions acquire operational relevance. The results presented in this section describe the consolidation of membrane-focused evidence retrieved through structured rotor architecture, the identification of recurrent routing-dependent mechanisms, and the formalization of membrane-level interpretative axes derived from the retained corpus. These findings remain confined to experimentally demonstrable membrane dynamics and do not extend into higher-order architectural synthesis.

#### 3.2.1 Global Membrane Architecture as a State-Dependent Regulatory Interface

Across the 112 retained studies (from 260 pre-deduplicated records across the three analytical rotors), convergent evidence indicates that the plasma membrane operates as a state-dependent, reconfigurable regulatory architecture rather than merely a passive structural boundary. Cholesterol- and sphingolipid-enriched microdomains repeatedly emerged as spatial control layers that determine whether receptors, channels, and signaling complexes can assemble, remain surface-competent, and transmit signals with defined amplitude and persistence.

Perturbation of raft integrity through cholesterol depletion, altered lipid flux, enzymatic remodeling, or genetic manipulation consistently produced predictable rewiring of downstream pathways. These shifts were observed across domains including innate immunity, angiogenesis, epithelial repair, metabolic regulation, and neuronal homeostasis. Importantly, in many contexts total receptor abundance remained unchanged, while signaling amplitude varied according to microdomain recruitment status. This pattern supports membrane organization as an independent decisional variable.

Mechanistically, three recurrent processes emerged consistently across independent experimental systems. First, regulated compartmentation and clustering of receptors within microdomains determined whether high-gain signaling states were enabled or suppressed. Second, microdomain-coupled trafficking programs adjusted surface density and topological availability of key proteins through endocytosis, recycling, shedding, or extracellular-vesicle export. Third, reversible lipid-dependent anchoring mechanisms linked membrane composition to voltage-, ligand-, or adaptor-dependent responsiveness.

In several experimental systems, membrane reconfiguration persisted beyond removal of the initiating stimulus or required active lipid restoration for reversibility. These observations are consistent with a membrane-level form of state persistence, suggesting that compositional reorganization can function as a short-term regulatory memory.

This global synthesis justifies a physiology-first interpretation: membrane composition, nanodomain organization, and trafficking competence collectively define an adaptive interface that assigns decisional weight to competing signals by controlling where, when, and for how long signaling components interact.

The analytical gradient of WP3 reflects increasing mechanistic specificity. Rotor I captured correlations between electrochemical membrane states and adaptive threshold behavior. Rotor II isolated cholesterol-enriched microdomain dynamics associated with receptor recruitment and signaling modulation. Rotor III retained experimentally validated routing mechanisms demonstrating membrane-dependent acquisition of transcriptional competence by exogenous nucleic acids.

The reduction from 260 to 112 studies therefore represents hierarchical refinement from descriptive membrane dynamics to experimentally validated routing competence. WP3 remains strictly confined to human cellular physiology and does not extend into theoretical claims beyond the mechanistic evidence retained.

#### 3.2.2 Structural framing of membrane integration through fundamental points of view

To maintain architectural continuity with WP2, the WP3 synthesis was structured using Fundamental Points of View (FPV’s) that formalize membrane-level decisional variables (Table 6).

**Table 6:** Presents the FPV framing derived from the nucleocapture–routing evidence block.

Structural axis (FPV)	Conceptual definition	Anchor evidence logic (refined)	Authors
Membrane-Governed Signal Access	The plasma membrane regulates whether exogenous nucleic acids acquire biological competence through structured capture and routing	Surface-associated nucleic acids require membrane-dependent internalization and compartmental access for productive IRF3/IFN signaling; ligand presence alone is insufficient when routing modules are disrupted or bypassed	Kleinman et al. (29), Obermann et al. (38), Singh et al. (31), and Wang et al. (102).
Microdomain-Dependent Routing	Lipid-associated adaptor modules couple surface clustering of ligands to endocytic trafficking toward PRR-permissive compartments	Recruitment of PRRs and adaptor complexes into lipid rafts or caveolin/flotillin-associated domains governs nucleic-acid uptake and downstream signaling amplitude	Watanabe et al. (30), Fork et al. (32), Liu et al. (103), and Kim et al. (104).
Compartment-Defined Signal Conversion	Transcriptional output depends on successful delivery of nucleic acids to specific intracellular sensing compartments	Endolysosomal localization, cholesterol-dependent membrane remodeling, and adaptor recruitment establish signal-permissive compartments; disruption of endosomal routing or nucleic-acid processing attenuates IFN or inflammatory output	Chen et al. (35), Ebihara et al. (105), Sun et al. (33), Tan et al. (36), and Perego et al. (34).

This framing operationalizes membrane architecture in terms of capture, routing, and compartmentalization rather than invoking generalized notions of raft “fluidity” or abstract membrane instability.

#### 3.2.3 Emergent critical success factors in nucleic acid integration

Inductive synthesis across the nucleic acid–focused rotor identified recurrent membrane-dependent control points that transcend individual receptor types. These are formalized in Table 7.

**Table 7:** Emergent critical success factors from nucleocapture–routing evidence.

Mechanistic construct (CSF)	Functional meaning	Evidence pattern	Authors
Nucleocapture Module	Specialized membrane-associated proteins enable binding and escort of exogenous nucleic acids	Loss-of-function blocks nucleic acid internalization	Obermann et al. (38), Singh et al. (31), Wang et al. (102), Watanabe et al. (30), and Inoue et al. (106).
Clustering-to-Trafficking Coupling	Surface clustering is insufficient; productive signaling requires coupling to endocytic machinery	Ligand clustering without uptake yields attenuated signaling	Kleinman et al. (29), Fork et al. (32), Ebihara et al. (105), and Yang et al. (107)
Microdomain-Linked Adaptor Recruitment	Lipid-associated scaffold proteins translocate upon stimulation and define routing pathways	Stimulus-dependent adaptor redistribution	Watanabe et al. (30), Fork et al. (32), Sun et al. (33), and Yang et al. (107).
Endosomal Competence Threshold	Signal intensity depends on delivery to PRR-positive compartments	Compartmental colocalization required for IFN output	Chen et al. (35), Ebihara et al. (105), Tan et al. (36), Perego et al. (34), and Ding et al. (108).
Routing Failure Dampens Signal Priority	Disruption of routing modules alters innate immune output without altering ligand availability	Reduced transcriptional response despite surface ligand presence	Fork et al. (32), Sun et al. (33), Perego et al. (34), Teo et al. (109), and Huang et al. (110).

These CSF’s do not describe receptor activation per se. They describe membrane-governed gating variables that determine whether exogenous genetic material becomes biologically effective.

#### 3.2.4 Mechanistic indicator set for membrane-level routing competence

To preserve cross-WP continuity, WP3 extracted a focused Mechanistic Indicator Set capturing operational membrane variables. Table 8 formalizes these indicators.

**Table 8:** Mechanistic indicator set extracted from nucleocapture–routing evidence (MIS-WP3).

Indicator family	What it captures	Operational readout	Conceptual bridge	Authors
Surface Clustering vs. Internalization	Distinguishes ligand binding from routing competence	Clustering without endocytosis in adaptor-deficient cells	Separates sensing from signal competence	Obermann et al. (38), Singh et al. (31), Wang et al. (102), and Inoue et al. (104)
Adaptor Translocation	Stimulus-dependent membrane recruitment	Cytosol-to-membrane redistribution	Event-driven membrane plasticity	Chen et al. (35), Sun et al. (33), Yang et al. (107), and Teo et al. (109)
Endocytic Coupling	Temporal interaction with trafficking machinery	Adaptor–clathrin association	Converts clustering into transport	Watanabe et al. (30), Fork et al. (32), Ebihara et al. (105), and Smythies et al. (111)
Compartmental PRR Colocalization	Delivery to signal-permissive organelles	Ligand–PRR endosomal colocalization	Compartment-based decisional logic	Chen et al. (35), Tan et al. (36), Perego et al. (34), Ding et al. (108), and Vidakovics et al. (112)
Transcriptional Output Dependency	Functional validation of routing	Suppressed IFN output upon routing failure	Architecture–gene regulation coupling	Kleinman et al. (29), Watanabe et al. (30), Fork et al. (32), and Koarai et al. (37)

The MIS-WP3 clarifies that membrane reorganization is not inferred indirectly from cytokine changes. It is operationalized through recruitment dynamics, cholesterol dependency, trafficking competence, and compartmental conversion.

#### 3.2.5 Integrative interpretation of exogenous nucleic acid routing

Across multiple experimental contexts, exogenous nucleic acids did not operate as autonomous signaling entities; rather, productive interferon induction required successful cellular uptake and endosomal delivery, as failure of membrane-associated routing modules abolished downstream IFN-*β* responses despite ligand presence (29, 30). In several systems, adaptor proteins transiently assembled capture-and-escort modules at the membrane, linking ligand clustering to trafficking toward signal-permissive compartments (30–32).

Disruption of microdomain integrity—through cholesterol depletion, efflux modulation, lipid remodeling, or genetic silencing—consistently impaired productive internalization and attenuated transcriptional output despite ligand presence (32–34). These observations indicate that routing competence, rather than ligand abundance alone, is a primary determinant of downstream signaling amplitude in nucleic-acid–sensing pathways, as cholesterol-dependent microdomain organization and endocytic coupling were required to convert surface-associated ligands into transcriptionally competent endosomal signaling (32, 33). Importantly, experimental systems in which ligand binding occurred in the absence of effective trafficking demonstrated reduced interferon induction, reinforcing the distinction between surface sensing and compartmental signal conversion (29, 30).

Collectively, the retained corpus supports the interpretation that exogenous nucleic acids acquire regulatory relevance conditionally, through membrane-level organization governing signal access, clustering competence, and compartmental conversion. Structural studies further demonstrate that recruitment of adaptor complexes within endolysosomal compartments establishes a decisive threshold for transcriptional activation (35), while lysosomal degradation pathways can modulate signal persistence by restricting nucleic-acid availability (36).

The regulatory impact of exogenous genetic inputs therefore appears to be mediated by membrane-dependent thresholding and trafficking architecture rather than by intrinsic ligand properties alone. WP3 remains confined to experimentally demonstrable membrane and endocytic dynamics and does not advance theoretical claims regarding distributed genomic participation. The membrane-centered synthesis presented here constitutes the structural substrate upon which subsequent cross-domain integrative analysis in WP4 is performed, remaining strictly bounded within mechanistic evidence retained under the predefined analytical scope.

### 3.3 Integrative pattern emergence

WP4 examined whether structural correspondences identified independently in WP2 and WP3 converge under predefined cross-domain integration criteria. The results presented in this section describe the alignment of functional stabilization patterns with membrane-level routing mechanisms and the formulation of explanatory models derived strictly from mechanistic convergence. No interpretative claims beyond the empirical boundaries established in WP2 and WP3 are introduced.

#### 3.3.1 Convergence of functional adaptation and membrane organization

WP2 indicated that adaptive physiological stabilization under exogenous genomic exposure manifests recurrently as immune recalibration, barrier gating drift, metabolic reprogramming, and microenvironmental constraint reinforcement. However, WP2 also revealed a structural asymmetry: while these adaptive outcomes are robustly documented at organ-system levels, the physical interface through which such adaptations acquire decisional weight remained comparatively under-synthesized.

WP3 independently identified membrane microdomain organization as a determinant of signal access, routing competence, and amplitude modulation. Across multiple human-cell models, Toll-like receptor responsiveness, adaptor recruitment, and downstream transcriptional output were shown to depend on cholesterol-rich membrane domains, caveolin/flotillin-associated scaffolds, and regulated endocytic delivery to signal-permissive compartments.

When these two evidence streams are analytically aligned under predefined convergence criteria, a conservative explanatory formulation becomes structurally permissible (Model A): adaptive physiological recalibration is operationally mediated through membrane-governed signal prioritization. Within this formulation, membrane microdomains function as threshold regulators that determine whether exogenous signals—metabolic, microbial, inflammatory, or nucleic-acid–derived—acquire biological competence.

Thus, immune setpoint reweighting (MIS-WP2) becomes mechanistically grounded in receptor clustering dynamics, lipid composition, and trafficking bias. Barrier plasticity is reframed not merely as transcriptional reprogramming but as membrane-topology modulation affecting ligand exposure and receptor accessibility. Metabolic reprogramming intersects structurally with membrane lipid flux, converting biochemical shifts into altered pattern-recognition sensitivity.

At this stage, no distributed genomic governance is inferred. The membrane is therefore positioned as a central routing substrate that mediates signal weighting within established physiological systems.

Importantly, the terminology introduced in subsequent sections reflects a deliberate shift from describing host–microbiome interactions as unidirectional influences toward a structurally integrated system perspective. While much of the literature characterizes microbial and environmental inputs as modulatory factors acting upon host physiology, the convergent evidence presented here supports an interpretation in which these inputs participate within a shared, membrane-mediated signal integration architecture.

In this context, terms such as “governance,” “weighting,” and “decision-making” are employed to denote the structured organization of signal prioritization across endogenous and exogenous inputs, rather than to imply anthropomorphic agency. Their use is intended to capture the transition from influence-based descriptions to an integrated, state-dependent physiological organization grounded in mechanistic convergence.

#### 3.3.2 Spatial encoding of barrier and interface plasticity

WP2 identified barrier plasticity (CSF-1) as a recurrent adaptive construct across inflammatory, ecological, and metabolic contexts. WP3 clarifies that such plasticity is spatially encoded within membrane-domain organization.

Multiple studies demonstrated that receptor activation—particularly for nucleic-acid–sensing PRR’s—requires inducible recruitment into lipid rafts and delivery to defined endosomal compartments. Conversely, disruption of cholesterol organization or caveolin/flotillin scaffolding shifts routing pathways and attenuates transcriptional output without altering ligand presence.

This convergence suggests that interface selectivity is not static but dynamically encoded in membrane topology. The plasma membrane does not merely separate intra- and extracellular compartments; it organizes decisional hierarchies by controlling access routes, clustering competence, and compartmental signal conversion.

Within Model A, barrier gating drift is therefore interpreted as membrane-level routing bias under adaptive conditions.

#### 3.3.3 Metabolic flux as gain control of signal sensitivity

WP2 consolidated metabolic reprogramming (CSF-3) as a recurring adaptive driver, particularly within lipid and redox pathways. WP3 extends this finding by demonstrating that cholesterol flux and oxysterol dynamics directly modulate pattern-recognition receptor signaling amplitude, linking membrane lipid availability to downstream transcriptional responsiveness (33, 36, 37).

Experimental modulation of cholesterol availability—through depletion, efflux activation, oxysterol synthesis, or pharmacological inhibition—consistently altered TLR responsiveness, p38 MAPK activation, and cytokine output (33, 37). In several systems, cholesterol enrichment amplified nucleic-acid–driven signaling, whereas cholesterol efflux dampened receptor recruitment into microdomains and attenuated interferon induction (36).

These findings establish metabolic reprogramming as structurally inseparable from membrane organization. Lipid flux does not merely accompany immune adaptation; it modulates receptor clustering competence and threshold sensitivity within membrane microdomains. In Model A, metabolic shifts therefore function as gain-control mechanisms acting through membrane-domain remodeling rather than as independent transcriptional drivers.

#### 3.3.4 Conditional competence of exogenous nucleic acids

WP2 identified explicit exogenous genomic interaction (FPV III) as structurally plausible but under-consolidated. WP3 provides direct mechanistic grounding for this layer.

Across multiple contexts, exogenous nucleic acids acquired biological efficacy only when membrane-dependent capture and routing modules were intact. Cholesterol conjugation enhanced cellular uptake and endosomal delivery of immunostimulatory RNAs (38). Raft-dependent adaptor recruitment facilitated poly(I: C) internalization and TLR3 activation (30, 32). Conversely, disruption of microdomain integrity prevented interferon output despite ligand presence (29, 34).

These findings converge on a critical principle: exogenous DNA/RNA does not autonomously modify host regulatory states. It acquires decisional weight only when membrane architecture permits capture, clustering, and compartmental conversion.

Under Model A, the membrane is therefore the necessary substrate for conditional exogenous genomic participation.

#### 3.3.5 From membrane-encoded signal thresholding to conditioned distributed participation of exogenous multigenomic inputs

Up to this point, the integrative synthesis supports a conservative explanatory model (Model A): membrane microdomains function as adaptive routing nodes that regulate the prioritization, amplification, and compartmentalization of exogenous signals within established host regulatory networks. In this formulation, the plasma membrane operates as a structurally organized interface determining whether external inputs acquire biological competence through controlled clustering, cholesterol-dependent recruitment, and delivery to signal-permissive compartments.

Model A sufficiently explains differential signal gain, threshold modulation, and context-dependent immune recalibration without invoking additional theoretical layers.

**Figure 4:**
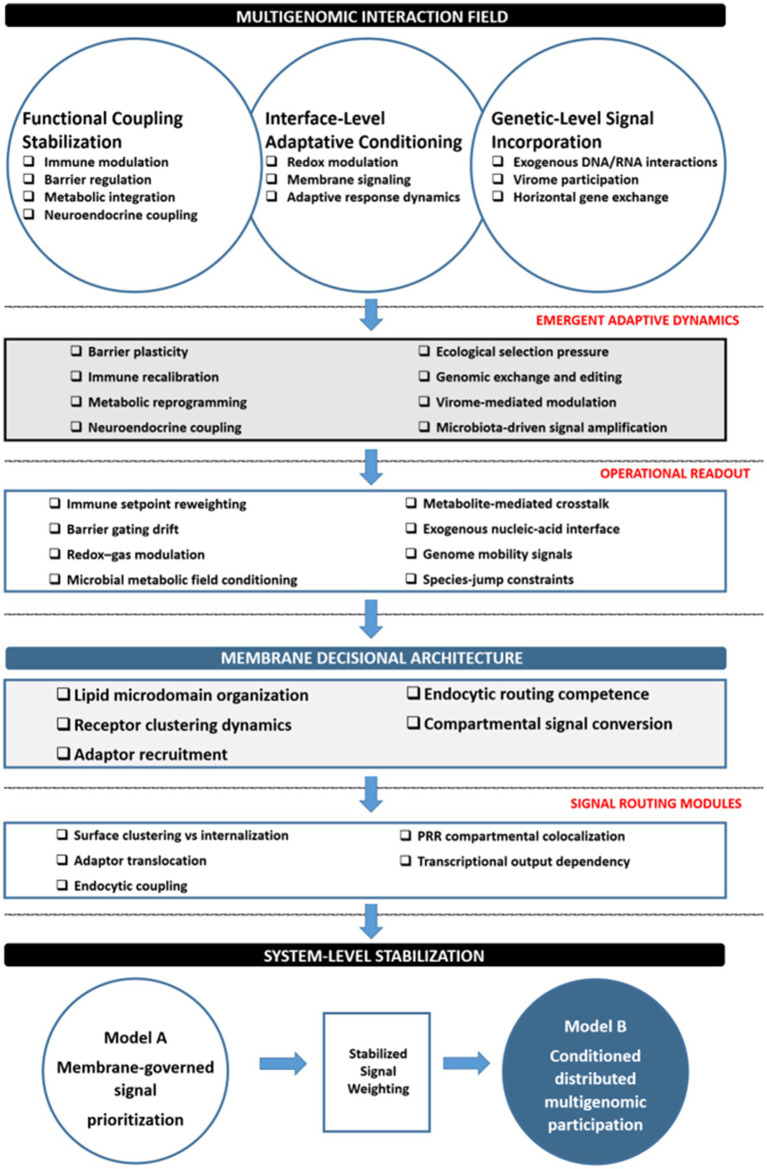
Membrane-level routing architecture linking ecological inputs to adaptive physiological stabilization within a multigenomic human system.

Note. This schematic summarizes the integrative architecture derived from WP2–WP4. At the ecological interface, diverse biological inputs—including microbial metabolites, immune mediators, redox–gas dynamics, metabolic signals, and exogenous nucleic acids—constitute a multigenomic interaction field organized across the Fundamental Points of View (FPV’s) identified in WP2. Cross-domain synthesis revealed recurrent stabilization behaviors formalized as Critical Success Factors (CSFs), including barrier plasticity, immune recalibration, metabolic reprogramming, ecological selection pressure, and microbiota-driven signal amplification.

The cross-domain convergence emerging from WP2 and WP3, and consolidated through the integrative synthesis conducted in WP4, is schematically represented in Figure 4. The diagram summarizes how multigenomic interaction fields, recurrent stabilization dynamics (CSF’s), and operational indicators (MIS) converge at the membrane decisional architecture, ultimately generating the system-level stabilization framework explored in the following sections.

These dynamics generate observable operational indicators captured in the Mechanistic Indicator Set (MIS), such as immune setpoint reweighting, barrier gating drift, redox–gas modulation, microbial metabolic field conditioning, and metabolite-mediated crosstalk. At the cellular interface, these indicators converge on membrane-level decisional architecture, where signal access and prioritization are governed through nucleocapture modules, microdomain-dependent adaptor recruitment, clustering-to-trafficking coupling, and endosomal routing competence (CSF’s identified in WP3).

These membrane-dependent routing processes produce operational readouts detectable through recruitment dynamics, endocytic coupling, compartmental colocalization, and transcriptional output dependency. Under typical physiological conditions (Model A), membrane-governed routing prioritizes signals within host-regulated adaptive responses. Under sustained ecological conditioning, stabilization of signal weighting may permit conditional multigenomic participation within the same membrane-governed decisional architecture (Model B), representing a structural extension grounded in cross-domain convergence rather than an autonomous regulatory layer.

WP2 demonstrated that long-term physiological stabilization recurrently involves explicit exogenous genomic participation, including extracellular vesicle–associated RNA’s, viral nucleic acids, and trans-kingdom small RNA’s. WP3 demonstrated that the biological efficacy of such nucleic acids is contingent upon membrane-governed capture-and-routing architecture. Importantly, membrane organization itself is dynamically modulated by metabolic flux, cholesterol redistribution, inflammatory signaling, and ecological conditioning. Moreover, lipid remodeling and adaptor recruitment can persist beyond the initiating stimulus in specific physiological contexts.

When these empirically grounded conditions are considered simultaneously, membrane-level routing modules cannot be interpreted solely as transient signal filters. They function as structurally embedded regulatory nodes that integrate endogenous and exogenous genomic inputs within the same physicochemical substrate.

This inferential extension does not imply teleological co-governance, nor does it attribute agency to exogenous genomes. Rather, it supports a constrained hypothesis (Model B): under conditions of sustained ecological exposure, multigenomic participation in long-term physiological stabilization may emerge as a structural consequence of membrane-governed signal routing.

In Model B, conditioned distributed participation of exogenous multigenomic inputs is not superimposed upon host regulation as an external or independent layer. Instead, it operates entirely through dynamically reconfigurable membrane interfaces that regulate signal access, clustering competence, and compartmental weighting across heterogeneous genomic sources. Membrane-encoded signal thresholding is therefore not only a mechanism of prioritization, but a structural prerequisite for the functional incorporation of exogenous multigenomic inputs into adaptive stabilization processes.

This extension remains strictly confined to the empirical boundaries established in WP2 and WP3 and is presented as a mechanistically grounded hypothesis rather than an ontological assertion.

#### 3.3.6 Integrative interpretation

Taken together, WP4 reveals structural convergence between adaptive physiological stabilization and membrane microdomain reorganization.

Functional integration (FPV I), adaptive dynamics (FPV II), and explicit genomic interaction (FPV III) are not independent strata but intersect at a common regulatory interface: the membrane-level decisional architecture. Immune recalibration, barrier gating, metabolic reprogramming, and nucleic-acid sensing repeatedly converge on shared membrane-governed variables, including receptor clustering, cholesterol-dependent recruitment, adaptor translocation, and compartment-specific routing.

The hierarchical asymmetry identified in WP2—robust consolidation of functional integration alongside fragmented synthesis of explicit genomic interaction—finds mechanistic clarification in WP3. The membrane interface provides the missing structural substrate through which exogenous genomic signals acquire conditional regulatory weight.

Model A establishes membrane-governed signal prioritization as a conservative explanatory framework. Model B proposes that sustained ecological exposure may, through the same membrane-governed architecture, enable distributed multigenomic participation in long-term stabilization.

The progression from Model A to Model B therefore reflects a structured inferential expansion grounded in cross-WP mechanistic convergence. Model A establishes membrane-governed signal prioritization as a sufficient explanatory substrate for context-dependent adaptive recalibration. Model B extends this architecture by recognizing that persistent ecological exposure may stabilize conditional multigenomic participation within the same membrane-routed decisional framework.

Crucially, this extension does not introduce new ontological assumptions. It formalizes a structural consequence of the empirically demonstrated coupling between membrane routing competence and exogenous genomic access. If adaptive stabilization repeatedly converges at membrane-level weighting mechanisms, then long-term physiological organization must be interpreted as emerging from hierarchical integration within that shared substrate.

### 3.4 Conceptual consolidation: symbiotic coherence governance in human biology

Given the structural convergence identified in WP4, the membrane-level decisional architecture described in Model A—and conditionally extended in Model B—can be interpreted as providing the minimal physical substrate through which multigenomic regulatory weighting becomes stabilized over time.

The conceptual consolidation presented here does not introduce an additional explanatory layer. Rather, it reorganizes the convergent findings of WP2, WP3, and WP4 into a systems-level interpretation consistent with the empirically bounded membrane-governed routing framework established above.

Within this consolidation, human physiology is interpreted as a symbiotic multigenomic system in which physiological states reflect stabilized hierarchical weighting of endogenous and exogenous regulatory influences operating within a shared physicochemical field. In this context, coherence denotes operational stability under a given decisional hierarchy rather than optimal functional performance.

Governance is defined operationally as the stabilization of hierarchical regulatory weighting within membrane-mediated decisional architectures. The term is used descriptively and does not imply intentionality, strategic agency, or competitive dominance among genomic entities.

The transition from an influence-based description of host–microbe interaction to a governance-oriented formulation was not introduced as a conceptual premise. It emerged progressively from cross-domain convergence between host–exogenous interaction layers mapped in WP2, membrane-level routing competence synthesized in WP3, and integrative consolidation criteria applied in WP4.

#### 3.4.1 The multigenomic human system

Although host and microbial cell numbers are broadly comparable (~3 × 10^13^ human cells versus ~3.8 × 10^13^ microbial cells), the asymmetry becomes more pronounced at the level of coding capacity (39). The human genome, currently estimated to contain approximately 19,000–20,000 protein-coding genes (40), represents a finite and relatively stable repertoire, whereas the collective metagenomic gene pool associated with the human microbiome contains millions of non-redundant genes with extensive metabolic versatility (41, 42).

This quantitative asymmetry becomes functionally relevant at biological interfaces, including epithelial barriers, immune surveillance zones, endocrine contact points, and membrane-level decisional substrates, where microbial metabolites, extracellular nucleic acids, inflammatory mediators, and environmental inputs exert measurable influence over host cellular responsiveness.

Within this framework, symbiosis is neither optional nor inherently beneficial; it constitutes a structural condition of human biology. Physiological states therefore cannot be defined by the mere presence or absence of exogenous influence, but by how decisional weighting is distributed within the integrated system and how coherence is maintained under that distribution.

#### 3.4.2 Decisional weighting and governance states

Physiological regulation is conventionally described through signaling cascades and feedback loops. While mechanistically accurate, this description overlooks a higher-order property revealed through WP4 convergence: hierarchical weighting of convergent signals. At any given moment, metabolic, immunological, microbial, neuroendocrine, and environmental inputs intersect under differential weighting within membrane-encoded routing architectures. The resulting physiological state reflects not the linear summation of these signals, but their relative prioritization as determined by membrane-governed thresholding and routing competence.

This hierarchical weighting is operationally observable as threshold modulation, receptor clustering bias, adaptor recruitment dynamics, and routing-dependent transcriptional amplitude, as demonstrated in WP3. Decisional weighting is therefore not metaphorical but structurally instantiated within membrane-level architectures.

Symbiotic coherence governance is defined here as stabilization of this hierarchical weighting over time. Under host-prioritized governance, symbiotic participation persists while systemic outcomes remain aligned with integrated organismal performance. Under displaced governance, coherence is preserved, yet decisional weighting shifts toward exogenous multigenomic inputs, localized cellular autonomy, or environmentally imposed constraints. Governance displacement does not imply loss of regulation; rather, it reflects stable reconfiguration of weighting hierarchies within the same membrane-mediated decisional framework. Chronic pathological states are frequently stable, reproducible, and resistant to perturbation, consistent with coherent biological organization operating under altered weighting regimes.

To preserve structural continuity between system-level manifestations and membrane-level operational mechanisms, the Mechanistic Indicator Sets derived from WP2 (MIS-WP2) and WP3 (MIS-WP3) were interpreted as complementary analytical layers within the consolidation process.

MIS-WP2 captures recurrent system-level features of adaptive stabilization across immune, metabolic, ecological, and genomic interaction domains, whereas MIS-WP3 resolves the membrane-level processes through which these features acquire signaling competence. This alignment indicates that observable phenomena such as immune recalibration, barrier gating modulation, and redox–metabolic conditioning correspond to specific membrane-dependent mechanisms, including receptor clustering, adaptor recruitment, endocytic routing, and compartmental signal conversion.

Within this consolidation, the relationship between these indicator sets is not hierarchical but translational. MIS-WP2 defines the observable system state, while MIS-WP3 specifies the structural constraints governing signal prioritization within that state. Their integration therefore provides a coherent cross-scale mapping between physiological manifestations and membrane-encoded decisional architecture, without introducing additional interpretative layers beyond the evidentiary boundaries established in WP2 and WP3.

#### 3.4.3 Cellular interfaces as decisional substrates

WP3 established that membrane microdomains function as conditional routing modules determining signal competence. Extending Model A, membrane architecture constitutes the physical substrate through which decisional weighting is implemented. The plasma membrane integrates chemical gradients, electrical potentials, redox states, lipid flux, mechanical forces, and trafficking competence. Modifications in membrane organization, including microdomain clustering, cholesterol redistribution, receptor recruitment bias, and endocytic routing, alter signal amplitude and compartmental conversion without requiring genomic sequence alteration.

Under hormetic conditions, such shifts modify responsiveness rather than enforce deterministic outcomes. Cellular behavior becomes context-conditioned rather than stimulus-determined. When membrane-level adjustments occur coherently across tissues, organism-level stabilization emerges without centralized command. Governance, in this sense, can be interpreted as distributed across cellular interfaces rather than localized in a single regulatory node.

#### 3.4.4 Canonical manifestations of governance displacement

Acute infection and sustained environmental toxic exposure can be interpreted, within this framework, as illustrative examples of temporary governance displacement. During acute infection, host physiology reorganizes metabolic allocation, immune prioritization, thermoregulation, and behavioral output in response to exogenous biological pressure. Coherence is preserved, yet functional weighting shifts to accommodate altered routing priorities within membrane-mediated architectures. Environmental toxic exposure similarly induces metabolic and immunological reorganization that may persist beyond removal of the initiating agent. In both cases, structural stability is maintained while hierarchical decisional weighting is transiently redistributed.

#### 3.4.5 Chronic disease as stabilized governance reconfiguration

Chronic diseases represent, in this consolidation, more slowly consolidated and deeply stabilized configurations of altered decisional weighting. The distinction does not reside simply in disruption, but in the duration, reinforcement, and systemic entrenchment of redistributed routing priorities within membrane-mediated architectures.

Across diverse chronic pathological states—including autoimmune disorders, metabolic syndromes, neuropsychiatric conditions, neurodegeneration, and cancer—recurrent stabilization patterns identified in WP2 reappear. Barrier gating drift, immune recalibration, ecological permissiveness, metabolic rigidity, microbial expansion, and localized cellular autonomy consistently emerge as system-level features. Conventional accounts interpret these phenomena as downstream consequences of inflammation, aging, genomic instability, or immunological dysfunction. From the perspective developed here, they can also be understood as manifestations of stabilized governance reconfiguration, in which hierarchical decisional weighting remains coherent yet persistently redistributed toward altered routing hierarchies, including heightened exogenous signal influence or localized subsystem optimization.

This articulation does not displace established etiological mechanisms nor assign autonomous agency to exogenous genomes. Instead, it describes a durable governance state characterized by sustained redistribution of regulatory influence within a shared physicochemical substrate, implemented through membrane-encoded signal thresholding and routing competence. The term “shared physicochemical field” refers strictly to the biophysical and biochemical environment defined by membrane composition, ionic gradients, redox balance, lipid flux, and trafficking dynamics, without invoking non-material constructs.

#### 3.4.6 Temporal consolidation and epigenetic stabilization

Time operates as a stabilizing axis within governance configurations. Repeated exposure to similar ecological constraints reinforces adaptive responses through epigenetic consolidation and membrane-level remodeling, processes widely described in trained immunity paradigms (43, 44). Aging-related disease progression can therefore be interpreted as progressive consolidation of governance states shaped by lifelong ecological interaction, consistent with established models of inflammaging and epigenetic drift (45).

Within this framework, epigenetics operates as a memory substrate of prior weighting regimes rather than as a primary etiological origin. Emerging evidence of trans-kingdom RNA exchange, extracellular vesicle–associated small RNAs, and coordinated shifts in stool miRNA and microbiome composition remains consistent with multigenomic integration, while remaining fully contained within the empirical scope established in WP2 and WP3.

#### 3.4.7 Operational manifestations across biological interfaces

The governance-based interpretation acquires practical relevance when examined across biological interfaces rather than isolated pathways. Stabilized decisional weighting is not confined to a single organ system but manifests across barrier tissues, endocrine loops, immune surveillance zones, and neurobehavioral outputs.

At mucosal and epithelial interfaces, altered membrane-level routing competence and ecological permissiveness can bias immune calibration and metabolic allocation without overt structural damage. At systemic levels, similar reweighting may manifest as persistent inflammatory tone, constrained metabolic flexibility, or altered neuroendocrine responsiveness. These manifestations do not reflect independent failures but coordinated stabilization within a shared decisional architecture.

Thus, the impact of symbiotic governance is not restricted to molecular interaction. It provides a structural explanation for why chronic phenotypes often exhibit cross-system coherence, resistance to isolated intervention, and non-linear recovery trajectories. The relevance of this perspective lies not in replacing mechanistic models, but in reorganizing them within a multiscale stabilization framework.

The structural consolidation established in WP4 defines the interpretative architecture. WP5 proceeds to examine whether this architecture remains coherent when expressed across classical physiological domains under physicochemical constraint.

## 4 Structural plausibility across physiological systems

WP5 was conducted as a structural plausibility assessment designed to determine whether the stabilization logic derived from WP2–WP4 remains internally coherent when expressed across classical physiological domains. Unlike WP2 and WP3, this stage did not involve systematic retrieval of new empirical material. Instead, it functioned as a consistency evaluation, examining whether the membrane-centered decisional architecture previously identified remains compatible with established principles of metabolic regulation, redox dynamics, autonomic modulation, acid–base balance, and barrier physiology.

The objective of WP5 was not to introduce additional mechanisms, but to test whether the integrative constructs emerging from cross-domain convergence remain physiologically defensible when situated within broader systems-level constraints.

### 4.1 Structured cross-domain triangulation procedure

WP5 operationalized structural plausibility through triangulation across three independently consolidated evidence domains, integrating membrane-level routing competence (WP3), functional stabilization patterns (WP2), and physicochemical as well as bioenergetic regulatory principles documented in previously registered systematic syntheses of microenvironment-dependent modulation.

Rather than introducing new empirical material, WP5 evaluated structural correspondence across these domains by examining whether stabilization patterns observed at the membrane level remain compatible with recognized principles of metabolic oscillation, redox coupling, proton dynamics, and mitochondrial constraint.

Triangulation was performed at the level of structural compatibility rather than causal inference. Constructs were retained when coherence could be demonstrated across at least two independent domains without requiring introduction of additional mechanisms.

### 4.2 Oscillatory stability under constraint

Previously registered and independently published systematic syntheses addressing microenvironmental regulation and bioenergetic stabilization provide an independent physiological backdrop against which the structural consolidation of WP2–WP4 can be evaluated (46, 47). These peer-reviewed reviews consolidate evidence that metabolic, redox, and proton dynamics operate within constrained yet dynamically adaptive ranges rather than fixed equilibria. Across tumor microenvironments and controlled physicochemical modulation contexts, mitochondrial coupling efficiency, extracellular acid–base regulation, and bioenergetic oscillatory behavior shift under sustained conditioning while preserving bounded regulatory coherence. Collectively, these syntheses demonstrate that physiological stability is better understood as constrained adaptive modulation rather than static equilibrium, thereby providing an independent systems-level reference frame compatible with the membrane-centered decisional architecture derived in WP2–WP4.

This alignment does not extend the empirical base of the study, but demonstrates that the governance-oriented interpretation remains structurally compatible with independent syntheses of physicochemical and bioenergetic stabilization mechanisms.

### 4.3 Stabilization under ecological conditioning

When the extracellular microenvironment is persistently conditioned by continuous inputs originating from the integrated multigenomic ecosystem—such as microbial metabolites, exogenous nucleic acids, inflammatory mediators, and redox-modifying signals—membrane-level decisional architecture may stabilize under sustained constraint.

In such configurations, regulatory weighting may cease to be anchored exclusively to human-centered functional optimization and instead converge toward preservation of coherence within the composite multigenomic system. This does not imply external control or intentional displacement of human regulatory processes, but reflects stabilization of decisional hierarchies within a composite biological system whose operative stability criteria extend beyond the human genome alone.

Under these stabilized regimes, membrane routing mechanisms can maintain systemic coherence even when organism-level functional performance becomes constrained. Clinical phenotypes may therefore manifest as acute infectious states or chronic pathological configurations without representing regulatory breakdown. In both cases, the defining feature is stabilization under an altered decisional hierarchy rather than loss of organization.

Comparable stabilization patterns have been extensively documented across non-human symbiotic systems, where host–partner interactions manifest as durable reweighting of functional priorities under ecological constraint (48–51).

### 4.4 Persistence, resistance, and reconfiguration

Clinical persistence phenomena—including post-infectious syndromes, chronic inflammatory patterns, metabolic rigidity, and therapeutic refractoriness—are structurally compatible with stabilization of routing-dependent decisional architectures rather than continued dominance of initiating factors. Such persistence patterns are widely documented in contexts of trained immunity and durable inflammatory memory (43), inflammaging and age-associated chronic immune activation (45), metabolic inflammation and loss of adaptive flexibility (2), and failure of resolution pathways in chronic inflammatory states (1).

Elimination of proximal contributors does not necessarily dissolve the stabilized configuration if membrane-level thresholding and feedback integration remain intact. Substitution phenomena, relapse trajectories, and resistance patterns are therefore consistent with preserved systemic coherence under altered regulatory weighting.

Within this perspective, pathology arises not from absence of regulation but from stabilization under a decisional configuration whose preserved coherence no longer aligns with host-prioritized functional optimization.

### 4.5 Distributed coherence across interfaces

Membrane organization integrates metabolic state, redox tone, lipid dynamics, inflammatory signaling, and ecological inputs into routing competence at the cellular interface. When such organizational variables stabilize coherently across tissues, coordinated systemic behavior may emerge without centralized command.

Under sustained ecological conditioning, the unit of preserved coherence may extend beyond isolated host cellular performance toward maintenance of the integrated multigenomic configuration. Acute infection and chronic disease can thus be interpreted as distinct temporal expressions of the same structural principle: stabilization of decisional weighting under altered environmental and symbiotic constraint.

WP5 therefore supports structural plausibility of the governance-based interpretation by demonstrating compatibility between membrane-level stabilization logic and established physiological principles. The analysis remains confined to empirical substrate established in WP2 and WP3 and functions as boundary calibration prior to formal systems consolidation in WP6.

## 5 Formal systems integration

WP6 constitutes the formal integration layer of the structured research sequence. Whereas WP2 mapped hierarchical asymmetries in multigenomic integration, WP3 demonstrated membrane-level routing competence, WP4 structured cross-domain convergence, and WP5 established structural plausibility under physicochemical constraint, WP6 consolidates these findings into a unified systems architecture.

At this stage, no new empirical evidence is introduced. The objective is to formalize the minimal structural configuration capable of accommodating the convergent patterns identified across prior work packages. The analyses consistently converge on two interdependent substrates: membrane-level decisional architecture and bioenergetic state stabilization.

Accordingly, WP6 articulates a membrane–bioenergetic systems framework in which physiological states are interpreted as stabilized attractor configurations emerging from interaction between multigenomic signaling inputs and microenvironment-conditioned energetic constraints. Within this architecture, membrane organization governs signal access and routing competence, while mitochondrial bioenergetics defines the energetic amplitude and plasticity range within which decisional hierarchies stabilize.

Chronic pathological states are therefore interpreted not as regulatory collapse but as coherent yet constrained attractor regimes maintained through membrane-level thresholding, lipid remodeling, redox bias, and NAD^+^-dependent repair limitation. Stabilization reflects reorganization of hierarchical weighting under sustained ecological pressure rather than structural failure.

### 5.1 Bioenergetic attractor states in multigenomic systems

Biological systems operate as dynamically stabilized configurations of energy flow, redox balance, and signal integration. In systems-theoretic terms, such configurations correspond to attractor states—relatively stable regimes toward which a system converges under defined environmental constraints (10). Oscillatory regulation of metabolic and signaling pathways, long recognized in biochemical systems (52), reflects bounded dynamic stability rather than static equilibrium.

Within a multigenomic human organism, homeostatic coherence may be conceptualized as a bioenergetic attractor characterized by efficient mitochondrial respiration, flexible carbon dioxide handling, balanced redox oscillation, adequate NAD^+^ availability, preserved repair capacity, and high membrane-level decisional plasticity. Mitochondrial function has been repeatedly identified as a central integrative node linking energetic constraint to disease susceptibility and adaptive capacity (53). Under such conditions, oscillatory dynamics across metabolic, inflammatory, endocrine, and autonomic domains remain broad and reversible.

Sustained ecological conditioning—including dysbiosis, inflammatory persistence, nutrient flux alteration, circadian disruption, or toxic exposure—can progressively reshape this energetic landscape. Rather than precipitating collapse, these perturbations may induce convergence toward alternative attractors characterized by narrowed oscillatory amplitude, reduced redox flexibility, and constrained decisional plasticity.

The defining feature of a displaced attractor is not molecular extremity but restriction of adaptive range. The system remains coherent and energetically stable while progressively limiting reversibility. Chronic disease, within this framework, reflects stabilization within such constrained yet internally consistent attractor regimes.

### 5.2 Gas dynamics as early modulators of attractor transition

Gas-mediated modulation represents a rapid physicochemical layer through which ecological inputs influence bioenergetic configuration. Carbon dioxide, as an obligatory product of oxidative metabolism, participates directly in acid–base regulation and intracellular proton availability, thereby influencing mitochondrial coupling efficiency and redox balance (46, 54). Because mitochondrial ATP synthesis depends on proton gradient integrity across the inner membrane, even modest shifts in CO₂ handling can alter the energetic landscape within which cellular stabilization occurs.

Gasotransmitters such as nitric oxide (NO), carbon monoxide (CO), and hydrogen sulfide (H₂S) further interact with mitochondrial electron transport chain components and redox-sensitive pathways. NO and CO reversibly modulate cytochrome c oxidase activity, directly influencing respiratory flux and reactive oxygen species dynamics (55). Hydrogen sulfide has been shown to regulate mitochondrial respiration in a concentration-dependent manner, acting as both substrate and signaling modulator (56). These gas-mediated interactions operate upstream of transcriptional reprogramming and can rapidly reshape metabolic throughput.

Because gases diffuse freely across membranes and alter proton gradients, lipid organization, and oxidative tone, shifts in gas composition may precede overt genomic or epigenetic remodeling. Altered intracellular acid–base microenvironments and redox states can reshape the conditions under which mitochondrial coupling stabilizes, consistent with systematic evidence that bioenergetic behavior adapts dynamically under sustained physicochemical conditioning (47).

Within this framework, gas dynamics function as early modulators of attractor transition, influencing the energetic landscape before higher-order genomic consolidation occurs. Gas-mediated shifts therefore represent rapid-state modulators capable of biasing stabilization trajectories without necessitating immediate transcriptional restructuring.

### 5.3 NAD^+^-dependent repair and stabilization reinforcement

As oscillatory bioenergetic flexibility narrows, NAD^+^ availability may decline relative to metabolic and repair demand. Age-associated and stress-induced reductions in NAD^+^ pools have been consistently documented as contributors to mitochondrial dysfunction and reduced adaptive capacity (57). Because NAD^+^ functions as both redox carrier and signaling substrate, shifts in its availability directly influence energetic amplitude and cellular plasticity.

NAD^+^-dependent regulators—including the sirtuin family of deacetylases—coordinate mitochondrial biogenesis, genomic stability, and stress adaptation through coupling of metabolic state to transcriptional and post-translational control (58). Declining NAD^+^ availability or impaired recycling can therefore constrain repair efficiency, reduce epigenetic recalibration capacity, and reinforce previously stabilized regulatory configurations.

Persistent redox imbalance and reduced NAD^+^-dependent signaling have been shown to interact with DNA damage responses, inflammatory tone, and metabolic rigidity (59). Within this framework, stabilization is reinforced not through catastrophic dysfunction but through progressive consolidation of constrained repair capacity and narrowed energetic range.

Stabilization thus becomes self-reinforcing: as repair efficiency declines and oscillatory flexibility contracts, transition to alternative physiological states becomes increasingly energetically demanding. Reversal remains possible but requires disruption of consolidated bioenergetic constraints rather than simple removal of initiating stimuli.

### 5.4 Regime-stabilizing feedback nodes

Within stabilized configurations, feedback modules such as the senescence-associated secretory phenotype (SASP) can function as reinforcement nodes. Cellular senescence is characterized not only by cell-cycle arrest but by sustained secretion of pro-inflammatory cytokines, growth factors, matrix-remodeling enzymes, and redox-active mediators that reshape the surrounding microenvironment (60, 61). These secretory programs are capable of altering immune tone, extracellular matrix composition, and metabolic signaling at tissue level.

Rather than representing primary pathology in isolation, such feedback nodes may consolidate previously established decisional hierarchies. By modifying the extracellular signaling landscape, SASP-related networks can reinforce constrained energetic and inflammatory configurations, thereby reducing local plasticity and enhancing regime stability (62).

Within the present framework, these nodes function as systemic expressions of attractor consolidation—secondary stabilizers that maintain coherence within already reweighted regulatory architectures.

### 5.5 Clinical phenotype as multiscale readout

Governance stabilization within multigenomic systems is unlikely to be reducible to single biomarkers. Contemporary physiology recognizes that homeostasis is not a static equilibrium but a dynamically regulated, distributed process maintained through continuous adaptive recalibration across interconnected regulatory systems (63, 64). The most reliable integrative expression of attractor stabilization is therefore the coherent clinical phenotype.

Persistent symptom clusters resistant to pathway-specific correction, constrained oscillatory range across metabolic and inflammatory axes, and non-linear recovery trajectories constitute operational signatures of stabilized regimes. Such phenomena are consistent with models of adaptive regulation in which physiological systems maintain coherence under altered regulatory weighting rather than returning to prior baselines (63). In this sense, the organism-level phenotype reflects the stabilized configuration of multiscale integration rather than isolated pathway dysfunction.

Gas dynamics, NAD^+^-dependent repair limitation, membrane-level thresholding, and feedback reinforcement represent mechanistic layers within the architecture; none alone defines the regime. Stabilization emerges from coordinated multiscale interaction and is most faithfully observed at the organismal level, where systemic integration—not molecular extremity—determines functional expression (64).

### 5.6 Operational hypothesis extraction from the integrated framework

Application of the predefined operational derivation criteria to the integrated architecture resulted in the identification of a constrained set of system-level hypotheses linking structural determinants to measurable physiological outcomes.

Specifically, constructs retained for operational translation were required to demonstrate: (i) recurrence across independent analytical domains (WP2–WP4), (ii) compatibility with experimentally validated membrane-level routing dynamics (WP3), and (iii) consistency with bioenergetic and physicochemical stabilization constraints (WP5). This filtering process excluded constructs lacking cross-domain support or mechanistic grounding, ensuring that all derived hypotheses remained strictly bounded within the evidentiary substrate.

Under these constraints, five primary operational domains emerged:

First, membrane-level signal routing competence was consistently identified as a determinant of downstream signaling amplitude. Across convergent evidence, alterations in lipid microdomain organization and adaptor recruitment were associated with measurable differences in transcriptional output independent of ligand abundance.

Second, cholesterol-dependent microdomain recruitment and clustering dynamics emerged as modulators of receptor responsiveness. Evidence across immune, metabolic, and signaling contexts indicates that cholesterol flux and lipid remodeling influence the probability of receptor clustering and subsequent signal amplification.

Third, threshold-dependent modulation of receptor responsiveness was identified as a recurring feature of adaptive stabilization. Convergent data suggest that membrane organization and electrochemical gradients define activation thresholds, resulting in context-dependent signal prioritization.

Fourth, oscillatory range and reversibility of metabolic and inflammatory states emerged as system-level indicators of attractor stability. Reduced oscillatory amplitude and impaired reversibility were consistently associated with stabilized pathological configurations.

Fifth, persistence of adaptive configurations following removal of initiating stimuli was identified as a defining property of stabilized regimes. Evidence across domains indicates that membrane-level reorganization and bioenergetic constraints can maintain system coherence even after elimination of proximal triggers.

These domains do not represent independent mechanisms but interconnected operational expressions of the integrated membrane–bioenergetic architecture. Together, they define a set of experimentally addressable relationships between structural organization and system-level behavior, enabling investigation of state transitions, responsiveness to perturbation, and restoration of adaptive range.

**Figure 5:**
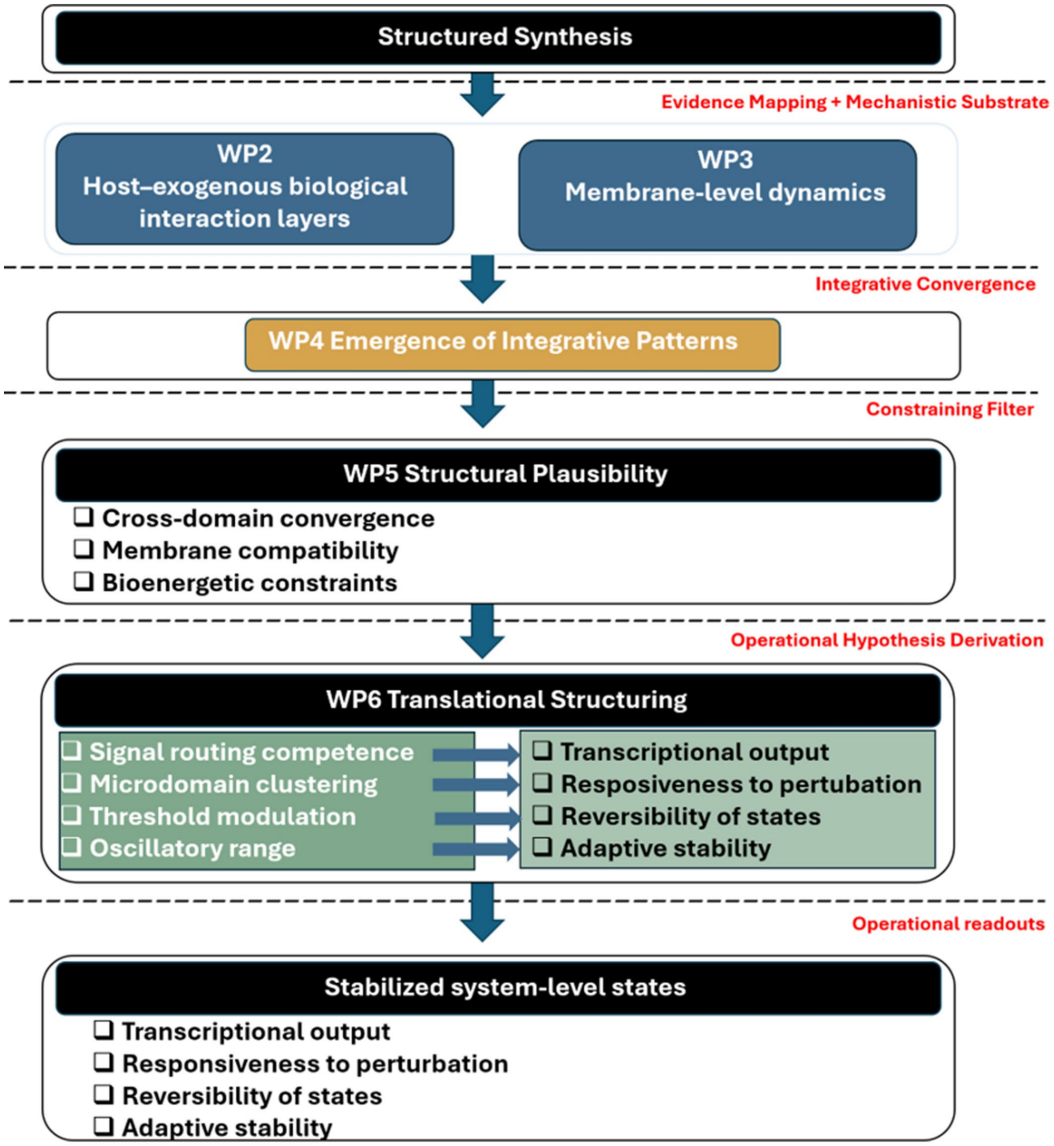
Operationalization pipeline of the multigenomic framework.

The translation of integrative constructs into experimentally tractable variables is conceptually illustrated in Figure 5 and operationally detailed in Table 9, which summarizes the identified domains alongside their corresponding structural determinants, membrane-level mechanisms, and measurable readouts.

**Table 9:** Operational domains and corresponding experimental readouts.

Operational domain	Structural determinant	Membrane-level mechanism	Measurable readout
Signal routing competence	Microdomain organization	Adaptor recruitment and routing	Transcriptional output (e.g., IFN signaling)
Microdomain clustering dynamics	Cholesterol-dependent membrane organization	Receptor clustering probability	Signal amplification / receptor activation assays
Threshold modulation	Membrane electrochemical gradients	Activation threshold regulation	Responsiveness to perturbation
Oscillatory range and reversibility	Bioenergetic flexibility	Redox–metabolic coupling	Recovery dynamics/state reversibility
Persistence of adaptive configurations	Stabilized membrane remodeling	Sustained routing bias	Maintenance of state after stimulus removal

Note. This schematic illustrates the methodological pathway through which the proposed framework is translated from structured evidence synthesis into experimentally tractable hypotheses.

The process begins with structured synthesis layers (WP2–WP3), comprising host–exogenous biological interaction mapping and membrane-level dynamics, which together define the mechanistic substrate of the system. These layers are integrated through cross-domain convergence (WP4), enabling the emergence of recurrent stabilization patterns across physiological domains.

Subsequently, a constraining filter (WP5) is applied, restricting interpretation to constructs supported by (i) cross-domain convergence, (ii) compatibility with experimentally validated membrane-level dynamics, and (iii) consistency with bioenergetic and physicochemical constraints. This step ensures that all derived constructs remain bounded within the evidentiary substrate and avoids interpretative extrapolation.

Following this constrained integration, the framework is translated into operational domains, including membrane-level signal routing competence, microdomain clustering dynamics, threshold-dependent modulation of responsiveness, oscillatory range, and state persistence. These domains are further linked to experimentally measurable system-level readouts, such as transcriptional output, responsiveness to perturbation, reversibility of states, and adaptive stability.

Together, this pipeline defines a structured transition from integrative synthesis to operational hypothesis derivation, enabling investigation of state-dependent physiological organization, stability, and transitions within a multigenomic human system.

### 5.7 Translational structuring: SMART and FINER alignment

To enhance conceptual tractability and research usability, the consolidated architecture was evaluated against the SMART (19) and FINER (20) criteria, which are widely used methodological heuristics for assessing the clarity, feasibility, novelty, and translational relevance of research questions and conceptual frameworks in biomedical investigation.

Table 10 presents the alignment between the proposed governance-based conceptual framework and these criteria, illustrating how the model satisfies established standards for specificity, measurability, feasibility, relevance, and methodological rigor in translational research design.

**Table 10:** Alignment of the proposed governance-based framework with SMART and FINER criteria for translational research design.

Criterion	Governance-based interpretation	Operational justification
SMART framework		
Specific	Focuses on identifiable state-dependent properties of physiology, particularly membrane-level decisional architecture, signaling thresholds, and oscillatory constraints	Research questions are framed around identifiable physiological states (e.g., loss of plasticity, hysteresis, biased responsiveness) rather than diffuse disease labels
Measurable	Emphasizes dynamic system properties rather than single biomarkers	Measurability derives from changes in reversibility, oscillatory range, responsiveness to perturbation, and persistence after stimulus removal
Achievable	Relies on established experimental and translational models	Existing in vitro, animal, and human physiological models already assess membrane biophysics, signaling sensitivity, immune tolerance, and metabolic flexibility
Relevant	Addresses clinically observed phenomena resistant to current explanatory models	Directly engages with chronic persistence, relapse after improvement, treatment resistance, and non-linear recovery trajectories
Time-bound	Incorporates temporal dynamics of stabilization and reversibility	Focuses on transitions between acute, adaptive, and stabilized states over time rather than static snapshots
FINER framework		
Feasible	Does not require new technologies or paradigms	Builds on existing methodologies in physiology, immunology, microbiome research, and systems biology
Interesting	Offers a unifying interpretation across fragmented biomedical domains	Integrates evidence from infection, inflammation, cancer, neuropsychiatry, and aging under a shared organizational logic
Novel	Introduces governance as an organizational hypothesis, not a new mechanism	Novelty lies in reframing known mechanisms as components of stabilized physiological states rather than isolated causal failures
Ethical	Explicitly non-prescriptive and non-interventional	Does not propose treatments, diagnostic criteria, or experimental manipulation beyond established ethical standards
Relevant	Aligns with unmet needs in chronic disease research	Provides a framework to generate testable hypotheses where guideline-concordant approaches show limited durability

Importantly, this alignment does not constitute empirical validation, but rather a structured methodological appraisal of the conceptual coherence and investigational viability of the framework.

Within this perspective, governance is formalized not as a diagnostic label or therapeutic prescription, but as an organizational physiological hypothesis capable of generating state-oriented research trajectories grounded in established principles of systems physiology.

WP6 completes the structural integration initiated in WP2. The membrane–bioenergetic axis is formalized as the shared substrate through which multigenomic signaling inputs, microenvironmental constraint, and adaptive stabilization converge into coherent physiological states. Chronic pathology is reframed as stabilization within constrained attractor regimes that preserve systemic coherence while limiting adaptive freedom. The architecture does not introduce new mechanisms; it reorganizes existing ones under a unified systems-level configuration.

## 6 Discussion

The preceding sections presented structured evidence consolidation and formal systems integration derived from WP2–WP6. The Discussion does not introduce additional empirical material, but interprets the implications of this integrative architecture within the broader context of contemporary biomedical reasoning. The aim is to clarify how membrane-level decisional stabilization and multigenomic integration may reorganize prevailing interpretations of chronic disease persistence, therapeutic refractoriness, and physiological coherence.

The interpretations presented in this section are derived from structured integrative synthesis and are intended as explanatory frameworks rather than definitive causal assertions.

### 6.1 From mechanistic fragmentation to organizational coherence

The present study does not propose a new mechanism, pathway, or therapeutic doctrine. Its central contribution lies in reorganizing convergent mechanistic evidence into an integrative architectural interpretation of human physiology. Across WP2–WP6, independent lines of systematic evidence revealed a consistent pattern: adaptive physiological stabilization under ecological pressure is mediated through membrane-level decisional architecture and reinforced through bioenergetic constraint.

Modern biomedicine has achieved remarkable precision in identifying molecular pathways and cellular effectors. However, its dominant explanatory model remains structurally reductionist, implicitly assuming that disease reflects localized dysfunction within a primarily host-genome–driven system. This assumption becomes increasingly strained when confronted with chronic conditions characterized by persistence, relapse, partial responsiveness, and resistance to pathway-specific intervention.

The data synthesized here support an alternative framing. Human physiology operates within a multigenomic ecological field in which host genomic processes coexist with diverse microbial and viral genetic repertoires capable of influencing metabolic, immunological, and signaling behavior (50). While recent quantitative revisions have corrected earlier exaggerations regarding microbial cell counts, the functional genomic asymmetry remains profound. The human genome encodes approximately twenty thousand protein-coding genes (40), whereas the gut microbiome alone encodes millions of genes with metabolic, enzymatic, and signaling potential (41, 42). Functional influence in biological systems is therefore determined less by cellular abundance than by encoded biochemical repertoire and environmental responsiveness.

Within such a system, regulatory stability cannot be reduced to host genomic programming alone. Physiological states emerge from weighted integration of endogenous and exogenous signals operating within shared physicochemical constraints. The key variable is not the presence of microbial influence, but how decisional weighting is distributed within the system’s regulatory architecture.

This reframing does not imply microbial dominance or teleological agency. Rather, it recognizes that multigenomic coexistence generates a shared decision space in which membrane-level organization, metabolic flux, and microenvironmental constraints determine which signals acquire functional priority.

### 6.2 Governance as stabilized hierarchical weighting

Throughout WP2–WP4, a consistent structural asymmetry was identified. Functional host–microbial integration is extensively documented at organ-system levels, yet the physical substrate through which signal prioritization is enacted remained comparatively under-synthesized. WP3 addressed this gap by demonstrating that membrane microdomains, lipid-dependent routing, and cholesterol-modulated receptor recruitment function as organizing interfaces that determine signal competence and signaling amplitude (65).

The integrative convergence of these findings supports a governance-based interpretation. In this framework, governance does not denote intentional control but stabilized hierarchical organization of regulatory influence within a multigenomic system. At any given moment, metabolic, immunological, microbial, neuroendocrine, and environmental signals converge within distributed regulatory architectures that prioritize certain inputs over others through threshold modulation and routing dynamics. Physiological states therefore reflect the relative precedence assigned to competing signals rather than their simple additive presence, consistent with contemporary views of biological regulation as distributed network organization (10).

Under host-prioritized governance, oscillatory freedom across metabolic and inflammatory axes remains broad and reversible. Under displaced governance, systemic coherence is preserved, yet decisional thresholds narrow and adaptive range becomes constrained. Chronic pathological states are therefore reframed not as regulatory collapse but as stabilization within alternative attractor configurations, in which the system maintains internal coherence while operating under altered weighting conditions.

This interpretation helps clarify a long-standing clinical paradox: many chronic diseases exhibit persistent energetic organization and structural stability despite adverse functional outcomes. Stability in biological systems does not necessarily imply health; rather, it reflects successful systemic organization under prevailing regulatory constraints (64).

### 6.3 Nutrition–microbiota–immunity: from axis to state architecture

Recent comprehensive reviews have meticulously mapped mechanistic links between nutrition, microbiota composition, immune calibration, and metabolic outcomes (66–68). These works have significantly advanced understanding of metabolite-mediated signaling, microbial modulation of nutrient bioavailability, and immune differentiation patterns across disease contexts. Microbial metabolites—including short-chain fatty acids, tryptophan derivatives, and secondary bile acids—constitute key mediators through which gut microbial communities influence host immune and metabolic regulation (69, 113).

However, these syntheses remain largely embedded within a disruption–correction paradigm. Chronic pathology is implicitly framed as imbalance to be normalized. What remains underexplored is why altered configurations persist as coherent, energetically stable states even after initiating perturbations are modified.

The governance-based framework extends existing knowledge by shifting the analytical unit from axis to state architecture. Nutritional inputs do not enter a neutral system awaiting correction; they enter a stabilized physiological configuration whose decisional architecture determines substrate allocation, metabolic routing, and immunological interpretation. This perspective helps explain why nutritional optimization may yield disproportionate or inconsistent functional recovery. The limiting factor may not be substrate availability but the prevailing governance state that filters and redirects inputs.

Thus, the contribution of the present framework is organizational rather than mechanistic. It does not replace established microbiota–immunity literature but provides an explanatory scaffold for understanding the persistence, stability, and resistance of physiological states over time.

### 6.4 Immune behavior as defense of coherence

In a governance-based interpretation, the immune system is no longer conceptualized solely as a binary detector of foreignness but as a subsystem that contributes to preserving systemic coherence under prevailing regulatory conditions. Contemporary immunological theory increasingly recognizes that immune responses are shaped by contextual signals reflecting tissue state, damage, and environmental conditions rather than simple discrimination between self and non-self (70, 71). From this perspective, immune activation, tolerance, or quiescence may reflect alignment with an established physiological configuration rather than direct expression of host-centered defensive intent.

Chronic low-grade inflammation, immune tolerance shifts, and paradoxical immune suppression can therefore be interpreted as coherent responses within displaced governance regimes. In autoimmune phenomena, immune reactivity may represent conflict between competing regulatory programs rather than intrinsic immunological malfunction. In oncological contexts, immune quiescence may reflect stabilized decisional weighting that deprioritizes surveillance within a coherent but host-suboptimal attractor state.

This interpretation is consistent with emerging concepts of disease tolerance, in which immune responses preserve organismal function and systemic stability even when pathogenic agents are not eliminated (72). Within this perspective, immune behavior participates in maintaining systemic organization rather than acting exclusively as a pathogen-elimination mechanism.

This reframing helps clarify why immune-targeted interventions frequently produce transient modulation without durable resolution. Unless membrane-level decisional architecture and bioenergetic constraints are renegotiated, downstream immune behavior remains aligned with the stabilized regime.

### 6.5 Eradication paradigms and stabilized states

Eradication-based strategies are indispensable in acute contexts where governance displacement threatens catastrophic loss of function. However, chronic symbiotically stabilized states present a different structural problem. Removal of a triggering agent does not necessarily restore a neutral baseline because no such baseline exists within multigenomic systems characterized by ecological complexity and microbial resilience (73, 74).

When eradication disrupts operational participants without reorienting decisional architecture, the system may reorganize to preserve coherence. Substitution phenomena, relapse patterns, antimicrobial resistance, and recurrence of dysbiosis are therefore consistent with ecological reconfiguration rather than simple restoration of a prior state. Stability is maintained; only its ecological composition shifts.

This interpretation does not diminish the importance of antimicrobial or anti-inflammatory strategies. Instead, it clarifies their scope. Elimination may remove the initiating perturbation, but durable resolution requires renegotiation of the decisional architecture through which physiological states stabilize.

### 6.6 Multigenomic governance beyond human systems

Symbiotic governance is not unique to human biology. Across ecological and evolutionary contexts, hosts and symbiotic partners frequently exhibit stabilized physiological reprogramming that preserves internal system coherence while supporting the persistence or propagation of non-host genomic cycles. Such dynamics are widely documented in host–microbe evolutionary biology, where organisms function as integrated ecological units rather than genetically isolated entities (49, 50).

Viral host reprogramming provides a particularly clear boundary condition within human biology itself. During infection, compact exogenous genomes can redirect host transcriptional, metabolic, and immunological resources toward viral replication while preserving short-term cellular viability and organizational integrity (51). These states illustrate how cellular systems can remain functionally coherent while decisional priorities shift toward supporting non-host genomic processes.

The framework proposed here generalizes this principle beyond acute infection, suggesting that certain chronic disease phenotypes may represent stabilized governance configurations rather than simple breakdown of regulation. The novelty of the present work therefore lies not in identifying a previously unknown biological phenomenon, but in rendering this organizational layer explicit within human physiology.

Importantly, this interpretation does not attribute cognition or intentionality to exogenous genomes. Governance emerges from encoded functional capacity interacting with environmental constraints within shared physicochemical fields. It is a systems-level property of biological organization rather than an expression of agency.

### 6.7 Limitations and scope boundaries

This framework is conceptual and synthesis-driven. It does not claim monocausal attribution between specific exogenous genomes and defined diseases, nor does it propose diagnostic criteria or biomarkers. Governance is inferred from sustained shifts in regulatory weighting, not directly measured.

Stability described here is not equated with benign outcome. Coherence may coexist with clinically adverse consequences. Host-derived processes—epigenetic regulation, aging, metabolic constraint—are recognized as co-determinants of stabilized states.

The hypothesis that certain ecological configurations may incidentally favor exogenous persistence under defined energetic constraints remains empirically testable rather than assumed universal. The framework proposes structural plausibility, not inevitability.

### 6.8 Research implications

The principal value of this work lies in expanding the space of legitimate scientific questions. Rather than targeting isolated pathways, research may increasingly examine state transitions, oscillatory amplitude, hysteresis dynamics, and membrane-level threshold modulation. Reversibility, responsiveness to perturbation, and restoration of adaptive range become operational variables.

This perspective does not prescribe therapies. It reorganizes fragmented evidence into a coherent architecture capable of generating testable hypotheses across molecular, cellular, systemic, and ecological scales.

By reframing chronic disease as stabilization within adaptive multigenomic regimes shaped by microenvironmental and membrane-level constraints, the governance model invites rigorous investigation of how physiological states are established, maintained, and potentially renegotiated over time.

At the membrane level, this perspective enables explicit experimental interrogation of signal prioritization mechanisms. For example, cholesterol-dependent microdomain organization may be investigated through controlled lipid raft perturbation and its impact on receptor clustering and downstream transcriptional output. Likewise, routing competence—defined by adaptor recruitment and endocytic coupling—can be assessed through compartmental colocalization assays and stimulus-dependent transcriptional responses.

These examples do not represent prescriptive experimental designs, but illustrate how the proposed framework delineates a tractable interface between membrane organization and measurable system-level outcomes, reinforcing its capacity to generate empirically testable research trajectories.

### 6.9 Translational implications

The governance-based framework proposed here is not intended as a therapeutic doctrine. However, it suggests several translational implications that may reshape how chronic physiological states are investigated and approached experimentally.

First, the framework emphasizes state transitions rather than isolated pathway perturbations as primary investigative targets. Chronic disease may therefore be more productively examined through measurements of oscillatory range, membrane-level signal thresholding, and bioenergetic flexibility rather than static biomarker deviations.

Second, the model suggests that restoration of adaptive range may represent a more informative therapeutic objective than elimination of individual molecular drivers. Within multigenomic physiological systems, removal of a perturbing agent may be insufficient if decisional architecture and bioenergetic constraints remain stabilized in a displaced configuration.

Third, the framework highlights the potential importance of membrane–bioenergetic interfaces as integrative control nodes. Membrane organization, mitochondrial coupling efficiency, redox balance, and NAD^+^-dependent repair capacity emerge as structural variables capable of modulating system-level responsiveness across domains traditionally studied in isolation.

Finally, the governance perspective encourages investigation of reversibility, hysteresis, and regime transitions as operational research endpoints. Rather than focusing exclusively on disease-specific mechanisms, future translational research may benefit from examining how physiological systems regain plasticity, expand oscillatory range, and renegotiate stabilized attractor states.

These implications remain exploratory. Their purpose is not to prescribe interventions but to broaden the conceptual space within which chronic disease persistence and physiological stabilization are investigated.

## 7 Conclusion

Health can be interpreted as the capacity of a multigenomic human system to sustain symbiotic coherence under host-prioritized regulatory weighting. In this configuration, membrane-level decisional architectures preserve adaptive flexibility, allowing coordinated oscillation across metabolic, autonomic, redox, and acid–base axes. Functional performance, resilience, and quality of life emerge not from static equilibrium, but from maintained oscillatory amplitude and reversible state transitions. Healing does not signify a return to a fixed baseline; rather, it reflects restoration of oscillatory freedom—the capacity to traverse physiological states without excessive energetic cost or defensive rigidity.

Recovery trajectories are inherently non-linear because stabilized configurations are historically reinforced. Physiological states consolidate through repeated ecological exposure, adaptive feedback loops, and epigenetic reinforcement. Biological systems therefore tend to revert toward previously established attractor regimes after partial improvement. Patterns of transient recovery followed by relapse need not indicate therapeutic failure; they may reflect persistence of an underlying decisional architecture. Durable improvement requires progressive stabilization of an alternative regime in which host-prioritized coherence becomes energetically sustainable.

This perspective reframes the role of medicine. Rather than focusing exclusively on eradication, suppression, or isolated pathway correction, clinical engagement may be understood as modulation of stabilized physiological regimes over time. The objective becomes support of adaptive transitions, prevention of reconsolidation into constrained configurations, and preservation of decisional plasticity within an ecologically integrated system. Mechanistic precision remains essential, but it operates within a broader multigenomic and physicochemical context.

The framework articulated here does not prescribe specific interventions. Its contribution lies in redefining the explanatory architecture through which chronic disease persistence and therapeutic refractoriness are interpreted. By conceptualizing pathology as stabilization within constrained attractor regimes shaped by membrane-level and bioenergetic conditions, future research can investigate how physiological contexts influence recovery of adaptive range and durable functional coherence. The advancement proposed is not a new doctrine, but organizational clarification—integrating molecular detail with systems-level stability across time.

## Data Availability

The datasets presented in this study can be found in online repositories. The names of the repository/repositories and accession number(s) can be found at: https://www.crd.york.ac.uk/PROSPERO.
